# The effect of sustainable mobility transition policies on cumulative urban transport emissions and energy demand

**DOI:** 10.1038/s41467-023-37728-x

**Published:** 2023-04-24

**Authors:** Lisa Winkler, Drew Pearce, Jenny Nelson, Oytun Babacan

**Affiliations:** 1 Department of Physics, Imperial College London, London, UK; 2 Grantham Institute – Climate Change and the Environment, Imperial College London, London, UK; 3 Transdisciplinary Studies in Global Change, Brunel University, London, UK

**Keywords:** Energy and behaviour, Energy modelling, Energy infrastructure

## Abstract

The growing urban transport sector presents towns and cities with an escalating challenge in the reduction of their greenhouse gas emissions. Here we assess the effectiveness of several widely considered policy options (electrification, light-weighting, retrofitting, scrapping, regulated manufacturing standards and modal shift) in achieving the transition to sustainable urban mobility in terms of their emissions and energy impact until 2050. Our analysis investigates the severity of actions needed to comply with Paris compliant regional sub-sectoral carbon budgets. We introduce the Urban Transport Policy Model (UTPM) for passenger car fleets and use London as an urban case study to show that current policies are insufficient to meet climate targets. We conclude that, as well as implementation of emission-reducing changes in vehicle design, a rapid and large-scale reduction in car use is necessary to meet stringent carbon budgets and avoid high energy demand. Yet, without increased consensus in sub-national and sectoral carbon budgets, the scale of reduction necessary stays uncertain. Nevertheless, it is certain we need to act urgently and intensively across all policy mechanisms available as well as developing new policy options.

## Abstract (web summary)

A rapid and large-scale reduction in car use, within a well-designed policy mix, is necessary to achieve short-term emission targets and reduce energy demand. Here, the authors introduce the Urban Transport Policy Model and demonstrate, using London as a case study, that current policies will not meet climate targets.

## Introduction

Cities are recognised as a key area for mitigation globally being responsible for 70% of global carbon emissions and consuming two-thirds of the world’s energy^1^. Cities have the potential to lead the way in the sustainable mobility transition; high population densities allow for short distances to be travelled between people and places and previously existing transport networks can be adapted to allow for multifaceted solutions combining infrastructural, technological, and behavioural change. Despite this, most cities in the world are still highly dependent on fossil-fuel based transit and are responsible for most transport-related emissions. In the UK, for example, the largest 20 urban areas are responsible for nearly 40% of the transport emissions of the UK (excluding international aviation and shipping)^2^.

Currently transportation accounts for 30% of global energy consumption, thus posing a challenge to the net-zero energy transition^3^. In the UK, electrification of the current car fleet requires an estimated additional 26.5% of the current electricity usage^4^. This extra demand alone is comparable to the entire current renewable generation capacity, highlighting the need to minimise demand in order to allow decarbonisation of the grid to continue at pace^4^. To accelerate this transition, policy intervention is needed and has potential to be highly effective^5^.

Although many policy decisions are made around local or national ‘zero-emission’ targets, such as the UK’s legally binding ‘net-zero’ by 2050 target, there is less pronounced policy discussion around meeting carbon budgets. These carbon budgets specify the remaining carbon emissions that can be released to have a “reasonable” chance of remaining within a 1.5 °C or 2 °C temperature rise, as set out in the 2015 Paris Agreement^6^. As such, emphasis should be put on whether cumulative emissions released between today and the future remain within this budget. This task is complicated by uncertainties in the climate models linking warming to carbon dioxide (CO_2_) emissions, variation due to different definitions of what is a reasonable chance of limiting warming to under a certain temperature and differences in agreements about what constitutes a fair share of the global budget for national and regional organisations. However, it remains clear that most organisations in the Global North will need to decarbonise as quickly or faster than the global average and this decarbonisation rate presents significant challenges.

Emissions can be mitigated in three fundamental ways: efficiency improvements, technological substitutions, and demand side solutions^7^. Whilst all three routes provide potential reductions in energy use, demand-side solutions allow for immediate reductions without needing to wait for deploying or developing new technologies^8,9^. As the target is a cumulative one, early savings cascade to have a greater effect than similarly sized but delayed interventions. Demand side solutions are often not prioritised in favour of electrification of existing technologies. National policy ambition in the UK, for example, proposes to meet climate targets by achieving only 9.5% through reducing demand^10^. This is because reducing energy demand in general is often associated with less economic growth, less social progress and inconvenient or drastic lifestyle changes^11^. However, for high-income countries in particular, policies which are designed to reduce energy demand whilst keeping economies stable and supporting social development can provide an immediate and cost-effective CO_2_ mitigation strategy^7,12,13^. Moreover, there are also co-benefits of reducing energy demand^14^; in the case of transportation, switching from cars to public transport would result in a net increase in employment^15^, increased population health^16,17^, as well as a reduction in reliance on imported energy^18^. Central to this is the concept of energy sufficiency, namely policies that aim to allow a level of energy use to sufficiently meet human needs^19,20^. Energy sufficiency can be seen as setting a minimum level of activity and helps outline a maximum level of ambition target.

Discussions are usually constrained by disproportionate attention to private car ownership, where the emissions and energy consumption of the car journey can be significantly reduced with EVs, but where this reduction is limited by the inherent energy required to power a small private vehicle at low occupancy. Given that just 1 in 50 new cars globally are electric and that cars have a lifetime of 15–20 years, it would take decades for the entire car fleet to transition away from fossil fuels^21,22^. In addition, the embedded emissions from vehicle production form a significant proportion of a vehicle’s life-cycle emissions and need to be accounted for when considering a switchover of the entire fleet^23^. On the other hand, a modal shift from cars to active travel or public transportation has the potential of reducing energy demand greatly thereby achieving short-term emission reductions cost-effectively^24,25^. This also leads to benefits such as decreased congestion, more space for communities, parks and walking or cycling routes, and less air pollution.

There is consensus in transport literature that electrification alone is not a viable solution and that a mix of policies is necessary^26–28^, but it is imperative for policymakers to understand how the policy mix interacts as a whole and whether individual policies complement one another or not^29^. For instance, policies promoting active travel and public transit options would reduce the abatement potential of car-related policies such as electrification of fleets or introducing new vehicles using other alternative fuel options. Thus, low-carbon transport policies would likely achieve stronger outcomes if assessed as bundles while considering their individual strong and weak points in delivering a sustainable mobility transition. Although there is wide-spread agreement that EVs emit less carbon over their lifetimes (even with current electricity emission intensities) than conventional internal combustion engine vehicles (ICEVs)^30^, there is debate about how large a role EVs should play in forming an exemplary sustainable city^31,32^.

Other policies aim to reduce the energy consumption from cars. For example, reducing the mass and size of cars (light-weighting) means less energy is spent on the average 1.6 passengers that occupy a 5-seater car^33^. Recent analysis has found that most SUVs in the UK are bought by people in cities^34^ and that increases in fuel consumption from larger cars have cancelled emission saving effects from EVs on a global scale^35^. Another policy includes converting an existing ICEV into an EV by replacing the drivetrain (retrofitting), which means no energy is spent on creating a whole new car chassis.

Many transport modelling frameworks exist to explore the impact of policies on emissions^36^. Notably, for the UK, TEAM^37^ is a sophisticated national transport model and has been developed to investigate energy demand reduction options for the UK^9,38^. Other studies in the UK have shown demand-side solutions and energy reduction options such as light-weighting to be imperative for near-term CO_2_ mitigation^39,40^. Transport models outside the UK have compared the impact of policies on emissions to national carbon budgets^26,41,42^ but have not explored the uncertainty in carbon budget allocation. Notably for urban analysis, a 2010 study analysed potential policy packages in London^43^ and more recently, CURTAIL was built as a transport modelling framework to analyse mitigation efforts for cities using Singapore as a case-study^44^. Nevertheless, there exists a lack of studies conducted at a local sub-national (e.g., city) level where policy suggestions become most relevant^45^ and this is especially true for assessments of sufficiency and energy demand reductions. This study aims to fill this gap by exploring a single regional sub-sector in high detail and comparing results to a wide range of carbon budgets. This is to assess the immediate actions that should be taken at a local level. We choose London as a representative urban-case study for the Global North to draw widely applicable implications to other similarly high-consuming and polluting cities.

In this study, we introduce a modelling framework called the Urban Transport Policy Model (UTPM) which models the future CO_2_ emissions and energy demand of the urban car fleet under several policy outcomes and we apply this model to London. Using UTPM we investigate the following question: assuming policy outcomes and behaviour change can be achieved, how far must we change the current trajectory of emissions in order to meet carbon budgets that align with the Paris Agreement? We do not explore how specific policies may impact choice, behaviour change and supply and demand, which can be found in other modelling frameworks such as TEAM^37^. Instead, we focus on overarching policy outcomes, including electrification, efficiency improvements and demand reductions assuming that they are equally feasible (with enough political drive). We explore how much of each intervention is necessary to meet different understandings of fair and sufficient carbon budgets for the Paris Agreement target of “well below 2 °C and pursuing 1.5 °C” including those agreed by different governmental organisations.

UTPM depicts the evolution of a passenger car fleet between 2020 and 2050 in which the transition to battery-electric vehicles (BEVs) and renewable energy is assumed and the effect of additional policies is investigated. The evolution of a car fleet year-upon-year is dependent on many factors, including the rate of adoption of BEVs, the rate of scrappage of ICEVs, and future travel demand. These parameters of the car fleet are often targeted by policymakers, who can provide incentives or set limits, taxes, and constraints on these parameters in order to achieve a certain policy outcome. Other parameters may not affect the specific make-up of the fleet but are important for emission or energy impacts. These include fuel efficiency improvements, weight reduction policies, and setting stringent emissions standards on manufacturing processes. Each of these parameters affect the fleet in at least one of the following three ways:

- Travel activity: the fleet size and how much the fleet is driven.
- Energy Iintensity of travel: energy consumed per vehicle kilometre driven.
- Emission intensity of energy: emissions released for every litre of fuel consumed or electricity spent.

The current trend of electrification and technological substitution means that most policies discussed today target the latter two parameters, which aim to improve the efficiency of car use^7^, whilst changes in travel activity are not equally well pronounced in regional and national decarbonisation pathways. To explore whether the current ‘improve’ policies are sufficient, we implement a wide range of these into the model, as described in Table 1. We investigate the importance of ‘avoid’ and ‘shift’ policies^7^ by exploring car travel activity exogenously. This policy outcome represents a myriad of policies that transform the car-dominated urban transport infrastructure into sustainable mobility infrastructure e.g., through substitution, modal shift, and distance reduction^5^. Combined into one parameter, we investigate the importance of the approach and encourage further work to study which policies can cause the demand reduction.

**Table 1:** List of policies considered and their details

Policy	Definition	Overarching policy outcome	Policy category
Phase-out	Setting a ban on the sale of new ICEVs by a certain date.	Electrification	Improve
Electricity decarbonisation	Transitioning to low-carbon electricity generation to power EVs with less CO_2_ emissions e.g. 100% renewable generation is considered.	Electrification	Improve
Retrofit	Converting an existing ICEV into an EV by replacing the engine.	Electrification, decrease in embedded emissions	Improve
Light-weighting	Encouraging the uptake of smaller, lighter vehicles. This can be incentivised by weight taxes or maximum weight mandates.	ICEV efficiency improvement	Improve
Scrap and replace	Scrapping an ICEV prematurely for a newer, more fuel-efficient ICEV, plug-in hybrid (PHEV), or EV which results in a faster turnover to EVs or more efficient ICEVs.	Acceleration of improve policies	Improve
Manufacturing standards	Setting strict manufacturing emissions standards on EVs or moving EV production to where these standards are in place to decrease embedded emissions.	Decrease in embedded emissions	Improve
Car travel activity reduction	Reducing the distance driven by cars as well as number of cars owned by switching transport modes to active travel (walking and cycling) or public transport.	Reduction in travel activity	Avoid/shift

We provide here the definitions of the policies implemented in the study, as well as their overarching policy outcome and policy category. All policies explored are ‘improve’ policies except for reduction in travel activity which represents a combination of ‘avoid’ and ‘shift’ policies.

The use-phase emissions in this study encompass emissions from the exhaust of ICEVs (also referred to as tailpipe emissions) within the geographical boundary of London and emissions from the life-cycle electricity generation for EVs. Since life-cycle values are considered for EV electricity generation, the use-phase emissions include emissions from building and using electricity generation and transmission infrastructure, EV charging points and battery storage for renewable energy futures. Well-to-tank (WTT) fuel emissions encompass fuel extraction, production, and delivery ‘to the tank’ for petrol and diesel fuel. The embedded emissions encompass the manufacture and end-of-life of vehicles and road infrastructure emissions such as road maintenance and construction. Use-phase and embedded energy demand follow the same respective definitions. Further assumptions are listed in the methodology.

We include the CCC’s 1.5 °C compatible pathway for surface transport as it is the current 1.5 °C scenario used for governmental policymaking and apply grandfathering principles to obtain the relevant proportion for cars in London. In addition, we include a more stringent estimate of the carbon budget for London from the Tyndall Centre^46^ due to its increased fairness approach, as it removes necessary global cement production and allows non “developed” nations to increase emissions to 2025. The remaining London budget is multiplied by the historical percentage of London’s emissions from cars^47^ (see Supplementary Note 1 for additional detail). The carbon budget considered in this study is directly comparable to tailpipe emissions only which occur within the boundary of London due to territorial-based carbon accounting methods. We also consider annual percentage reductions including the global 1.5 °C IPCC target of 45% emissions reduction by 2030 and net-zero in 2050^48^ and the 1.5°C lifestyles target which represents the UK emission reductions required for equal per capita global emissions in 2030^19^.

A baseline case provides a ‘business-as-usual’ reference point for the effectiveness of other policies to be compared to. The baseline case follows the historical trend for fleet size and distance driven (equating to a 20% increase from 2020 to 2050 in line with predictions from the UK’s Department for Transport^49^, a scrap age of 15 years (resulting in an average car attrition rate of 5% in line with the previous decade^50^), a 2030 phase-out for ICEVs and 2035 for PHEVs (as set by UK government), ‘net-zero’ electricity generation emissions in 2050, unregulated manufacturing standards for BEV production, and no significant retrofitting or light-weighting.

Three magnitudes of car travel activity reduction are investigated, each representing a different modal shift target from the current car modal share of 36%^51^. These are converted from modal shares to distance driven using a stochastic model based on the National Transport Survey, see Supplementary Note 2 in the Supporting Information for more details.

- Current Greater London Goal^52^: to reach a car modal share of 20% which we estimate means a reduction in car distance driven of 43%.
- Current City of Paris Goal: to reach a car modal share of 12% which we estimate means a reduction in car distance driven of 66%.
- A Stretch Goal: to reach a car modal share of 6.5% which we estimate means a reduction in car distance driven of 81%.

Emissions from other transport modes resulting from a modal shift are estimated by multiplying the reduction in car distance driven by the average life-cycle emissions intensities of non-car modes such as walking, cycling, using trains and busses. The percentage of travel by each mode and public transport occupancies are assumed to stay constant at London’s current levels and busses are assumed to be electric with emissions decarbonising at the rate of the electrical grid assumed in the model.

## Results

**Fig. 1:**
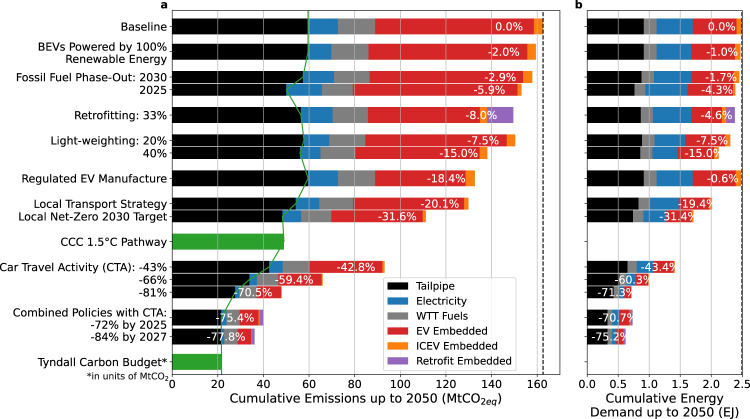
London car cumulative CO_2eq_ emissions and energy demand under different policies. Cumulative emissions (**a**) and energy demand (**b**) between 2020 and 2050 is shown for the baseline case and each policy applied onto the baseline case, as well as the local transport policy for London and a combined case consisting of all policies acting together. Emission projections are compared with the Tyndall Centre’s estimate of the carbon budget (for well below 2 °C and pursuing 1.5 °C)^46^ multiplied by the historical percentage of London’s emissions from cars^47^ and the CCC’s cumulative emissions pathway for 1.5 °C for surface transport^10^ multiplied by the historic percentage of UK surface transport emissions arising from London cars (see Supplementary Note 1). Emissions and energy demand are categorised by their source; the fossil fuel energy and tailpipe emissions from ICEVs (black), the electricity consumed for driving EVs (blue), the well-to-tank emissions from fuels (grey), the embedded emissions and energy from EVs (red) and ICEVs (orange) and the emissions and energy from retrofitting ICEVs with electric engines (purple). Note: the Tyndall carbon budget is in units of MtCO_2_ and does not account for other greenhouse gases, so it is a slight underestimate of the entire CO_2eq_ budget.

Figure 1a shows that the current system cannot reach stringent carbon budgets without adopting highly aggressive and disruptive policies. Electrification, including moving the phase out date forward, results in cumulative emissions 7 times greater than the Tyndall carbon budget for the “well below 2 °C and pursuing 1.5 °C” global temperature target. Rather, a combination of aggressive policies is necessary so that future emissions reach levels comparable to the carbon budget. Of these policies, the most important is reducing car travel activity. Policies that decrease car distance driven and car ownership by over 80% as compared to current levels are highly effective in edging close to the designated carbon budget.

The least effective policy outcome in our considered portfolio is faster electrification; advancing the full phase out of fossil fuel cars, including hybrids, by 2030 reduces cumulative emissions by less than 3%. Advancing this phase out to 2025 only results in an emissions reduction of 5.9% compared to the baseline policy that already assumes a phase-out date of 2030 for ICEVs and 2035 for hybrids. Thus, advancing the phase-out date forward by 5 years only affects new cars bought in this 5-year period. As this is a small percentage of the existing fleet and of all the cars manufactured in the period of 2020–2050, the emissions reduction potential associated with moving the phase-out date forward by 5 years is relatively small. Furthermore, whilst tailpipe emissions are decreased by 16%, the electricity emissions are increased by 19% for the 2025 phase-out case compared to the baseline case. Thus, without rapid decarbonisation of the electrical grid used to power EVs, emissions savings from early phase-out are counteracted by emissions from a largely fossil-fuel dependent electrical grid as well as emissions from EV manufacture.

Powering all BEVs by renewable electricity generation reduces emissions of travel using BEV by 30%. However, when considering tailpipe emissions from new and old ICEVs and embedded emissions from new vehicles, the overall change in emissions is just 2%. This is because emissions from electricity are small compared to tailpipe and embedded emissions. These emissions can be targeted by light-weighting policies which decrease all emission sources for new cars: tailpipe emissions, BEV electricity emissions, and embedded emissions from the decreased amount of material needed. Figure 1a shows light-weighting provides a fleet-wide emission reduction of around 8% for every 20% of mass of new ICEVs and BEVs decreased. It also has many additional benefits such as decreasing air pollution from car exhausts and tyres and decreasing motor vehicle related deaths^53^. Similarly, additional fuel efficiency improvements such as downsizing engines should further decrease emissions.

The latest local transport strategy for London is the Mayor’s Transport Strategy published in 2018 which aims to achieve a 12% reduction in distance travelled by car by 2041^52^. This strategy achieves a reduction in cumulative emissions from the baseline case of −20.1%. A recent report published for the Greater London Authority by Element Energy analysing the measures required for a net-zero 2030 target in London recommended a 27% reduction in vehicle kilometres relative to 2018 by 2030 for an accelerated green scenario^54^. However, this achieves a reduction in cumulative emissions of −31.6% and fails to meet the Tyndall carbon budget, with tailpipe emissions exceeding it by more than double. Nevertheless, this scenario meets the 1.5 °C pathway derived from the UK’s Committee on Climate Change (CCC) carbon budget^10^. However, the scale of system change that is required between a 27% and 80% reduction in car travel activity is a vast range. Therefore, agreeing on a carbon budget or an alternative framework for sectoral analysis within regions is imperative if policymakers are to have any clarity as to what is necessary to meet the Paris Agreement.

Embedded emissions of BEVs can be reduced by 42% through implementing stricter manufacturing standards. These regulations refer to the strict emissions standards that can be set on the manufacture of the batteries (responsible for a large proportion of a BEV’s lifecycle emissions and energy consumption due to the high shares of fossil fuels used in the regions where most battery cells are produced^30^) and other components of an electric vehicle. Thus, manufacturing cars with greater amounts of renewable energy as well as using less emissions intensive manufacturing processes such as recycled content and heat recovery, results in an overall cumulative emissions reduction of 18.4%. Policies should therefore incentivise the operation of EV battery recycling and manufacturing on a national level, where jurisdiction is possible over the energy sources and manufacturing practices used.

Retrofitting also provides a reasonable emission reduction of 8% when one third of scrapped fossil fuel cars are converted to EVs instead. This emissions reduction is expected to increase if EV conversions were to be applied to all cars, and not just scrapped cars as modelled in this study. However, retrofitting a large proportion of the ICEV fleet is currently not seen as viable as the development cost per vehicle type and cost of conversion for most vehicles is higher than manufacturing a new electric car. This is due to economies of scale of new electric vehicle production and design incompatibilities between conventional cars and electric engines. Yet, with the right incentives and government support to drive innovations, retrofits can become cheaper and more commonplace. These include creating manufacturing incentives to motivate businesses specialising in EV conversion, creating development incentives which help fund product development cycles for cost-effective conversion kits/designs and development of the EV servicing industry, tailored to handle ageing body maintenance and long-life electric drivetrains such as battery replacement and repurposing.

### Cars currently on the road cause majority of emissions

Tailpipe emissions (black bars in Fig. 1a) represent the largest proportion of cumulative emissions in the combined policy cases and a key area for mitigation. In the baseline case, 78% of these tailpipe emissions are caused by cars in the existing fleet made pre-2020 rather than new cars introduced in the period of 2020–2050. In the combined policies cases, 99% of the tailpipe emissions stem from the existing fleet. Hence, it is the exhaust emissions from cars on the road today living out their life that are responsible for 68% of the total emissions in the combined case and that are using up more than 92% of the carbon budget, even if disruptive policies are implemented. Therefore, policies solely focusing on reducing the carbon footprint of new cars cannot be sufficient in achieving climate targets without introducing regulations that target the existing fleet. This can be done through measures that encourage less driving (e.g., modal shift), retrofits of the existing fleet, fuel-saving driver behaviour (e.g., optimal speed travel, which is not modelled in this study) and potentially low carbon synthetic fuels, although their impact on emissions is not established.

Applying modal shift policies result in the most substantial emission reductions due to less car travel activity overall. The reduction in emissions with reduction in distance driven is approximately linear with a 43% and 81% decrease in car distance driven by 2027 resulting in a 42.8% and 70.5% decrease in cumulative emissions respectively. This applies when travel is avoided, however, when car transport is modally shifted to other transport modes, the energy demand and emissions released from those alternative modes are increased. Nevertheless, the resources needed for active travel and shared transport infrastructure are significantly less than private vehicle ownership^24,55^. Active travel also holds many co-benefits, such as the health benefits gained from physical activity and the freeing up of urban spaces from traffic thus creating safer and less polluted cities^16^. Whilst an 81% decrease in car kilometres results in a 70.5% decrease in cumulative emissions, some of this reduction will be offset by an estimated increase of 28 MtCO_2_ from the alternative transport modes, representing an additional two thirds of the cumulative emissions for that scenario. However, this estimate does not explore the full decarbonisation potential in non-car transport modes. For example, modal shift emissions are greatly reduced when public transport occupancies are increased and non-car modal shares are dominated by active travel. It also does not consider the light-weighting potential in buses or the influence electric micromobility may have.

In Fig. 1, we demonstrate two additional scenarios that meet the Tyndall carbon budget. Both require BEVs to be powered by 100% renewable electricity, a 2025 fossil fuel phase-out, 33% retrofitting, 40% light-weighting, strict standards on EV manufacture as well as a massive and rapid reduction in car travel activity. The scale of reduction necessary is either a 72% reduction in distance driven by 2025 or an 84% reduction in distance driven by 2027. This translates to just 1 in every 12 and 1 in every 24 trips made by car, respectively. Thus, waiting longer to act on car travel demand requires much more stringent policy measures in the future. This echoes the sentiment from the first UK climate assembly discussing net-zero transport; people would rather act earlier to avoid more drastic action later^56^. Similarly, people would rather remove the most carbon intensive cars from roads straight away for less people to have their car use constrained through pricing or physical restrictions in the future^56^.

Figure 1a also shows that the percentage emissions savings of each policy do not add linearly when applied together in the combined cases. This is because when all policies act simultaneously, the effects from one policy can often reduce or cancel out the effectiveness of other policies. For example, improvements in emissions intensities of cars become less impactful when combined with a large decrease in car travel activity. Thus, it is important to consider which policies are responsible for most emissions reductions within a specific combination of policies in order to give appropriate levels of resources to each policy.

Figure 1 shows a clear correlation between policies that reduce cumulative emissions most and those that reduce the cumulative energy demand most. If energy use were to remain relatively similar, emissions would need to be mitigated through introducing ‘emission-free’ technology. However, this introduction of new technology is energy- and emission-intensive itself, as shown by the large red embedded EV regions in Fig. 1. These regions can be targeted by retrofitting ICEVs with electric engines which avoids the need to manufacture new EVs, by light-weighting EVs which minimises material use and by modal shift policies which reduces the need to manufacture new cars altogether.

**Fig. 2:**
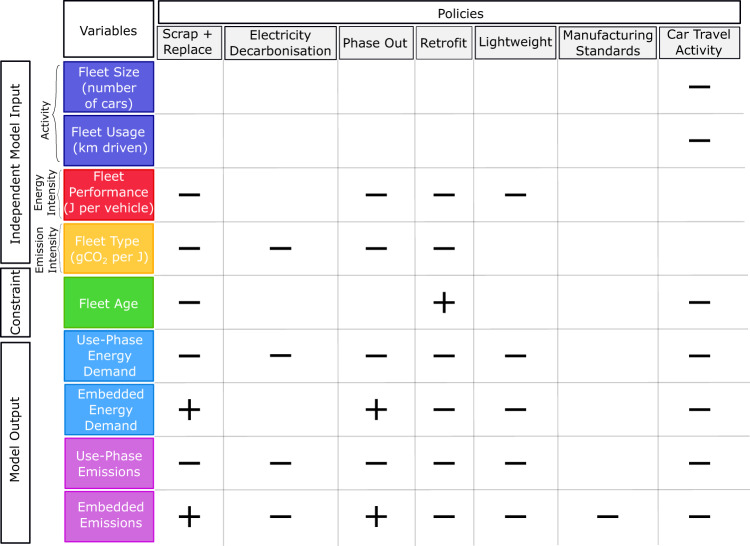
Effects of different policies within the UTPM. The policies can either increase (plus-sign) or decrease (minus-sign) the magnitude of variables, which are divided into inputs, constraints and outputs for the model. Fleet size refers to the level of activity of the fleet (size and usage), fleet performance refers to the energy efficiency per vehicle (or alternatively per km driven) and fleet type refers to the emissions intensity per Joule spent in the vehicle (which is dependent on the type of vehicle). Outputs are divided into energy demand (light blue) and emissions (purple). All policies decrease use-phase emissions, apart from manufacturing standards which decreases embedded emissions. Thus, each policy has potential to reduce fleet-wide emissions, and their behaviour and influence in the model is summarised here.

Figure 2 shows how the set of policies considered affect the fleet and its emissions and energy impacts. The plus or minus signs correspond to whether a policy results in an increase or decrease in magnitude of the corresponding variable. Increasing any of the variables increases emissions or energy demand, except for fleet age which is dependent on the specific make-up of the policies. This demonstrates the complexity and interdependence of the many policies and variables. For example, a policy can be helpful for decreasing use-phase emissions, but detrimental to embedded emissions, such as introducing EVs and the premature scrapping of ICEVs. In addition, implementing two or more policies that influence the same impacts in different ways can create new behaviour, so that a combination of two policies changes the effect a single policy could have. For example, premature scrapping and replacement of ICEVs decrease cumulative emissions without a modal shift as it promotes a faster transition to electric vehicles assuming additional EV demand can be met. However, when combined with a modal shift, premature scrapping can increase emissions as entire new electric vehicles are manufactured rather than retaining ICEVs for a few years until going car-free is possible. Thus, there is often a balance to be achieved when choosing policy options.

### Some policies are effective but too slow for climate impact

**Fig. 3:**
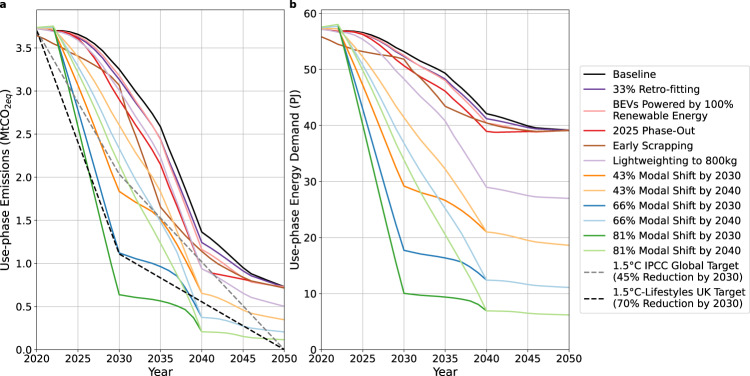
The impact of various policy options on use-phase emissions and energy demand. The 2020–2050 London car annual CO_2_ emissions (**a**) and energy demand (**b**) results are shown for the baseline case and different policy options applied to the baseline case. Six reductions in distance driven are explored, at three magnitudes of 43%, 66% and 81%, representing the London goal, Paris goal and a stretch goal, and at two different rates, until the year 2030 and 2040. The grey dashed line shows the IPCC target of 45% emissions reduction by 2030 and net-zero in 2050 in line with a global 1.5 °C degree target^48^. The black dashed line shows the 1.5 °C lifestyles target which represents the UK emission reductions required for equal per capita global emissions in 2030^19^.

Figure 3a shows the use-phase emissions of the baseline case and several policy options. Only a reduction in distance driven results in short term emission reductions that meet 2030 targets. Although all policies reduce emissions by a factor of 4 or more by 2050, the rate of emission reductions vary greatly. Policies that rely on the turnover of the fleet, henceforth called ‘turnover policies’, such as electrification and light-weighting, are too slow considering emissions globally need to be almost halved by 2030 in order to meet 1.5 °C carbon budget^48^ However, to reach equal per capita emissions globally in 2030, the UK needs to decarbonise faster reaching a 70% emissions reduction by 2030^19^.

Scrapping cars prematurely to accelerate the effects of turnover policies still does not achieve considerable reductions in short enough timescales, shown by the red line in Fig. 3a. The effectiveness of the reduction in car travel activity policy in reducing emissions is also dependent on the rate of implementation; reducing distance driven by 2040 does not reach short-term emission reduction targets.

In addition, none of the policies reach net-zero in 2050. This is because there are still emissions associated with renewable electricity generation in 2050, such as from the maintenance and construction of renewable energy generation and transmission infrastructure and its storage requirements. Thus, for car emissions to reach net-zero in 2050, these emissions would need to be mitigated through different measures such as using natural-based or engineered carbon dioxide removal measures. A large modal shift is the only way to reach as close to net-zero as possible.

Figure 3b shows the use-phase energy demand of the car fleet. Electrification of the fleet results in 30% less annual use-phase energy demand in 2050 than the fossil-fuel based fleet in 2020. On the other hand, car travel activity reduction and light-weighting policies result in significantly lower energy demands in 2050 than electrification and the rest of the policies. This is because the other policies all result in the same 2050 scenario: a same-sized 100% electric car fleet, with differing rates and methods of electrification to get there. It is this remaining energy demand resulting from electrification alone that would make it very difficult to reach close to net-zero in 2050 and would require significant renewable energy capacity to be built for the use of private vehicles.

### Combining policies to achieve climate targets

**Fig. 4:**
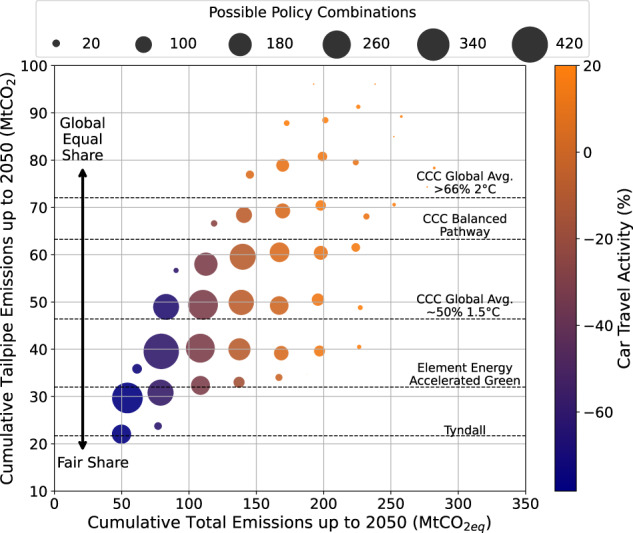
The relationship between cumulative tailpipe CO_2_ emissions and total CO_2eq_ emissions for different policy combinations. Cumulative tailpipe emissions and total emissions, including use-phase (tailpipe and EV electricity), WTT fuel and embedded emissions, between 2020 and 2050, are plotted for varying magnitudes of car travel activity, scrapping, light-weighting, retrofitting, ICEV phase-out, regulated EV manufacture and electricity decarbonisation policies. Magnitudes of modal shift are distinguished by colour with blue (dark) corresponding to a modal shift of −80% and orange (light) to a modal shift of +20% (baseline case). The results are grouped by number of policy combinations within that emissions range with the largest bubble corresponding to 400 distinct combinations of the policies and the smallest bubble to 2. Five carbon budgets are plotted along the tailpipe emissions axis (y axis) because the carbon budgets are limited to the boundary of cars in London and do not include emissions outside London, such as electricity generation emissions or car manufacturing emissions.

Figure 4 shows the tailpipe cumulative emissions against the total cumulative emissions (including electricity emissions from EVs, WTT emissions of fuels and embedded emissions of vehicles) of different combinations of policies we have analysed including car travel activity reduction, scrapping, light-weighting, retrofitting, ICEV phase-out, regulated EV manufacture and electricity decarbonisation grouped by number of policy combinations that reach that point in the phase space. The colour of the results corresponds to the average reduction in distance driven in the scenarios, with purple corresponding to a reduction of −80% (darkest) and yellow corresponding to a baseline travel activity increase of +20% (lightest). The size of bubbles corresponds to the number of policy combinations within a cumulative tailpipe emissions range of 10 MtCO_2_ and a cumulative total emissions range of 30 MtCO_2eq_.

When considering multiple carbon budgets, derived under different methods and allocation principles from the global carbon budget for 1.5 °C (see Supplementary Note 1), the amount of reduction in car travel activity that is necessary is uncertain. For example, taking the UK carbon budgets from the CCC (for a 50% chance of meeting 1.5 °C and considered lenient among other carbon budgets^57,58^) allows, in a small set of cases, for car travel activity to increase as ‘business as usual’, although this is at the expense of total emissions including electricity generation and embedded emissions. Therefore, to avoid emissions from car use entering other carbon budgets, car travel activity must be reduced significantly and urgently.

The exact degree of mitigation effort required in terms of reducing car travel activity is very sensitive to the carbon budget. For the combined policy case (assuming the highest mitigation efforts in electrification and fuel efficiency) and applying a reduction in car travel activity ranging from −50% to −90% by 2027 results in a cumulative tailpipe emissions range of 8 MtCO_2eq_ (see Supplementary Note 4 for additional details). Thus, at that range, an uncertainty in carbon budget of ±4 MtCO_2eq_ results in an uncertainty in car travel activity of ± 20%. Similarly, achieving a 50% reduction in car travel activity by 2025 rather than 2035 saves 6.5 MtCO_2eq_ of cumulative tailpipe emissions. This represents a high effort of mitigation for a relatively low amount of carbon, and thus it could be argued these carbon savings may be better made in a different sub-sector. Since there is no agreed consensus on regional and sub-sectoral carbon budgets, and uncertainties are large, it makes it difficult to ascertain exactly how far we need to go with reducing car travel activity.

### The risk in policies failing

Decreasing car travel activity narrows the distribution of emissions results, represented by a larger number of policy combinations within a smaller emissions range (bigger bubble) in Fig. 4. This is because at low levels of car travel activity, car-related policies such as light-weighting and the ICEV phase out become much less significant. As we have modelled them, these policies only affect new cars, so they would require a large influx of new cars to make a substantial impact on emissions. Similarly, as the distance driven is greatly reduced, retrofitting and electricity decarbonisation policies which target the use phase emissions of cars have a limited impact. Other policies such as light-weighting and electrification also work to localise emissions results (see Supplementary Note 5). Working toward a high-likelihood emissions reduction target that can be achieved through numerous distinct policy outcomes (larger bubbles) decreases the risk of an exact policy outcome or emissions target not being achieved. Reaching stringent carbon budgets, such as the Tyndall Centre’s, has a smaller set of policy combinations (smaller bubbles), so there is less room for error if policies fail. If regions are to set a carbon budget target, certainty on the cumulative emissions associated with policies is paramount, and so placing all bets on just one mitigation strategy, such as electrification or modal shift alone, leads to a greater risk of not achieving emissions reductions than implementing a carefully designed mix of policies. It also highlights the need that if we are to aim for a budget as tight as Tyndall’s, then we need to search for a wider set of policies or push policies further to increase the likelihood of reaching it.

One such risk is if decarbonisation of the electricity grid were to happen at a slower pace, the carbon reduction potential of electrifying the car fleet would be negatively impacted. This risk can be mitigated through a reduction in energy demand of the car transport system. The same cumulative emissions that can be achieved with ‘net-zero’ in 2025 can also be achieved with 3% less cumulative energy demand from cars and ‘net-zero’ in 2060 (see Supplementary Note 8). Thus, reducing energy demand from cars through modal shifts and policies such as light-weighting is a more effective carbon mitigation strategy than electricity decarbonisation for transport emission mitigation.

### Not all policies result in emissions reductions

Supplementary Note 6 explores the combination of policies in further detail. For instance, it shows that the car travel activity, light-weighting, retrofitting, ICEV phase-out and electricity decarbonisation policies are monotonic in the sense that a greater magnitude of the policy outcomes results in greater emission reductions. The scrapping policy, on the other hand, can result in greater emissions with too high and too low a scrap age depending on the wider policy landscape. This is because there exists a balance between use-phase and embedded emissions. Although scrapping cars earlier results in use-phase emissions savings it can be outweighed by an increase in emissions from building new EVs to replace functional ICEVs. This chimes in with other evidence which has shown scrappage schemes to save little carbon and potentially increase emissions on a life cycle basis^59^. Scrapping only becomes a feasible policy when it results in a replacement of an ICEV for an EV or a modal shift. In a wider context, a policy may be detrimental to emissions in one scenario and beneficial in another. Such interdependent relationships should be explored further when evaluating region-dependent policy options.

If a decrease in car activity is to be implemented, it is important to consider an effective pathway to the final car ownership target. A combination of policies that incentivise the purchase and uptake of EVs along with a simultaneous shift away from car infrastructure may result in ‘wasted’ embedded emissions. This is because EVs may be purchased and then remain largely unused or be scrapped prematurely for other modes of transport. Thus, modal shift policies should be planned according to a target number of cars in the city in the future in order to not create under-used excess cars in the meantime. We demonstrate this concept more in detail in Supplementary Note 7.

### Recommendations

Our analysis has shown that reducing car use is essential to meeting carbon budgets. However, current infrastructure in many cities renders this unfeasible as many people do not currently have the choice and the means to take sustainable forms of transportation. Thus, taking climate targets seriously opens a window of opportunity for an entire transformation of the current urban landscape to prioritise urban space development for people rather than for cars. We briefly discuss policies that can be implemented to target car travel activity:

- Car clubs allow users access to a vehicle without owning one and has been shown to reduce the number of car trips^60^.

- Car-free zones and car-free days can free up urban space to be transformed to green areas, markets and playgrounds which can lead to beneficial health effects and are likely to lead to higher levels of active mobility^61^.
- Dynamic road user charging is a road pricing scheme that considers geographical location as well as the local impacts of using the road to congestion, air pollution and noise^62^. In such a scheme, urban areas with access to many alternative modes of travel would see higher prices of using roads than rural areas, which in turn ensures low-income households pushed to car-dependent areas are not negatively impacted^62^. Previous natural experiments and studies have found road pricing schemes to reduce number of car trips^63,64^.
- An immediate halt in urban road building projects and existing roads repurposed to prioritise active transport, such as the Barcelona ‘Superblock’ model^65^. In London, this would include a halt to projects such as the Silvertown tunnel built to facilitate more river crossings in cars^66,67^.
- Better planning regulation to ensure new houses and communities can only be built with good transport links and services such as post offices, shops, health centres and schools^68^. The concept of the 15-minute city in which most daily necessities can be accomplished by active travel from homes has gained popularity and is being implemented in cities such as Paris^69^.
- Along with policies that discourage car use, significant improvements should be made in the accessibility, affordability and connectivity of public transportation.

UK climate assembly members voted in favour of most of the above interventions, however, only 17% of UK climate assembly members voted for the scenario which reduces car travel activity most^68^. Concerns around quality of life, especially for disabled communities, restricted choice, feasibility and cost were raised. Thus, reducing car use presents a significant social and behavioural challenge. Whilst extreme and disruptive policies are necessary to avoid worsening effects of climate change globally, current policy framing based on popular vote is not well suited to delivering drastic change in short timescales. Measures of ensuring the populace is brought on board with these changes is vital and need to be urgently developed.

An opportunity for further research exists in modelling car travel activity endogenously through policies such as implementing car-free zones, dynamic road user charging, car-sharing, micromobility, and more. Another opportunity lies in modelling policies that reduce the emissions intensities of ICEVs used at present, such as lower speed limits and behaviour change in driving style. This would help inform how to deliver the solutions required for an urban transport system that meets stringent carbon budgets.

## Discussion

A rapid and large-scale reduction in car use is necessary to achieve short-term emissions targets, meet stringent carbon budgets that limit temperature rise to 2 °C at most and to not create excessive demand for additional technology, material, and minerals. Our analysis demonstrates that relying on efficiency and electrification policies takes too long due to the turnover time of the fleet, even with the ban on the sale of fossil fuel vehicles moved forward and accelerated scrapping of ICEVs. This is because the vast majority of carbon emissions that fill up the remaining carbon budget originate from cars that already exist. Policies that can reduce these emissions include retrofitting the existing fleet with electric engines and reducing the distance driven by cars. Policy efforts should put increasing emphasis on mitigating emissions from cars that exist today rather than solely focussing on the electrification of new cars.

There is an inherent risk if policies fail that emissions reductions will not be achieved, and carbon budgets will not be met. This risk can be mitigated by combining many policy levers together, such as efficiency, electrification, and demand-side solutions, to reduce the total energy demand of the system. The effect of this is a narrowing of the distribution of potential future cumulative emissions and a higher certainty of achieving a given carbon budget. For example, with lower future energy demand from cars, there is less risk of a slowly decarbonised electricity grid increasing the cumulative emissions of transport. It also reduces the pressure on the electrical grid from EVs and allows for an easier switch to a renewable powered future across all sectors.

Meeting the Tyndall carbon budget requires a 72% reduction in car travel activity by 2025 as well as a fossil fuel car phase-out in 2025, 100% renewable generation for electric cars, retrofitting a third of scrapped fossil fuel cars with electric engines, light-weighting new cars by 40% and setting strict standards for EV manufacture. This would mean that only 1 in 12 trips can be made by car and would achieve a reduction in total cumulative emissions of 75.4%. If the rate of modal shift implementation is slower, then an 84% reduction in car travel activity is required by 2027 to meet this budget, allowing only 1 in 24 trips to be made by car. The magnitude of intervention necessary will be relevant to other cities with a high responsibility to reduce emissions from today’s levels, but we recognise the magnitude of implementation will vary by city. Our framework can be applied to other regions using the online UTPM modelling software (see the methodology for details).

This analysis provides insights into the effectiveness of various policy options in the urban sustainable mobility transition, but it has limitations. The ‘improve’ policies were modelled endogenously and based on concrete implementable policies such as a 2025 phase out date, whereas the ‘avoid/shift’ policy of reducing car travel activity was applied exogenously. Whilst this methodology means the reduction in emissions due to reduction in demand may be obvious, the main insight is the discussion of the magnitude of car travel activity necessary to meet regional carbon budgets, and the sensitivity these carbon budgets have on the demand reduction suggested by modelling exercises. This may explain why there is currently little consensus on the level of demand-side mitigation required within climate policy. It is clear from this work that urgent frameworks and agreements need to be developed on sub-national sectoral carbon budgets for regional policymakers in urban areas to direct serious and confident efforts at policies that meet the Paris Agreement. This conclusion is relevant irrespective of region, and other urban areas should apply this approach to explore how the effectiveness of their policy packages depend on carbon budgets.

## Methods

The Urban Transport Policy Model (UTPM) is an object-oriented python model, available online at https://github.com/LisaOJWinkler/UTPM. It is based on a model car fleet, initialised using city-specific data, undergoing an annual evolution consisting of old cars being scrapped and new cars being added to the fleet from 2020 to 2050. Although the model code is available online and can be applied to any region and year, the following method applies to London with a base year of 2019. The following paragraphs outline the methodology behind the model and model assumptions.

### Implementation of policies

Whilst the improve policies are mainly national and undeliverable by London on its own, we assume that these policies are implemented nationally and pushed forward on a local level through providing sufficient charging points, retrofitting centres, ensuring car dealerships sell electric cars only after the phase-out of ICEVs, local scrappage centres, and so on. Whilst consent for national policy is bred locally, councils may also not achieve goals without national policy, and so coproduction of local and national policy is assumed. The improve policies would achieve emissions reductions irrespective of region, however, the avoid/shift approach for reducing car travel activity is a local problem by nature. Local walking and cycling infrastructure, public transport links, land use management and urban planning, etc., are inherently local policy, and so we compare all policies in this study on a local, urban level.

### Car fleet initialisation

The initialisation of the car fleet is based on three data sources; total number of cars in London in 2019, *C*, the age distribution of cars in the national 2019 car fleet, *A*, and the annual historic proportion of new cars sold of each fuel type (adoption rate), *P*, all sourced from the Department for Transport’s vehicle statistics database (DfT)^50^. Changing these inputs allows the model to be applied to different cities. The number of cars in the 2019 fleet of a given age, *a*, and fuel type, *f*, is given by,

1 $${S}_{2019,a,f}=C\times {A}_{a}\times {P}_{f},$$

and every thousand cars with the same fuel and age attributes are created as objects in the model (at a resolution of 1000 cars per object to speed up processing time). Conventional hybrid cars are included under petrol cars. Alternative fuel cars are included under battery-electric cars. The list of inputs and a flowchart for the car fleet initialisation is provided in Supplementary Note 9.

### Car fleet evolution

An annual cycle is assumed where old cars are removed from the fleet and new cars enter the fleet. The number of new cars in year *Y* is given by,

2 $${N}_{Y}={S}_{Y}-{S}_{Y-1}-{O}_{Y},$$

where *S*_*Y*_ is the fleet size in year *Y* and *O*_*Y*_ is the number of cars scrapped in year *Y*. In reality, cars undergo a change of ownership where old cars are sold on a second-hand vehicle market. However, the overall effect is still that in any given year, new cars enter the car fleet and old cars leave the fleet, and so the simplified model approximates real behaviour.

The fleet size is specified as a policy parameter and depends on the magnitude and rate of a change in car travel activity. If implemented, the fleet size follows a linear increase or decrease until 2040 of magnitude specified by a percentage of the 2019 fleet size. If no modal shift is implemented, a ‘business-as-usual’ trend is assumed consisting of a 20% increase from 2019 levels in 2050. The scrap age is also specified as a policy parameter. Cars above a scrap age, *y*, are removed from the fleet. To avoid irregularities in the model where the introduction of the scrap age results in a large proportion of the fleet suddenly removed, a grandfathering scrap age can be specified for all cars manufactured pre-2020.

The number of new cars of a given fuel type *f* in year *Y* is given by,

3 $${N}_{Y,f}={N}_{Y}\times {P}_{Y,f},$$

where ${P}_{Y,f}$ is the adoption rate of the fuel type in year *Y*. The future adoption rates of each fuel type are based on assumptions given in Supplementary Note 10. The list of inputs and a flowchart for the car fleet evolution is provided in Supplementary Note 11.

### Low-carbon fuel source

In this study electricity is considered as the only low-carbon alternative to fossil-fuels in near-term as EVs are currently the only commercially proven alternative technology for passenger cars that are globally available in the consumer market. Moreover, based on current evidence, EVs require a lower energy budget for light-duty travelling and result in less emissions compared to ICEVs even if new ICEVs were to use bio-derived or synthetic fuel alternatives rather than fossil fuels^70,71^. Firstly, producing electricity renewably provides a sustainable source of low-carbon energy carrier (i.e., fuel in EVs). The carbon abatement energy defined as the net energy invested per net CO_2_-equivalent emissions mitigated is substantially lower for renewable electricity production compared to renewable hydrogen gas and synthetic hydrocarbon production^72^. Secondly, the evaluations for wheel-to-tank (energy spent and associated emissions to deliver the fuel from raw materials into the vehicle) and tank-to-wheel (energy spent and associated emissions to drive a set distance) performance of EVs are so far better than the alternatives even in countries with electric grids that largely rely on fossil fuels for electricity generation^73^. Finally, even when the increased embedded emissions due to battery manufacturing are considered, EVs at present achieve 65% lower life-cycle emissions than ICEVs over the average lifetime of a passenger car^74,75^.

### Distance driven

The annual distance driven per car is given by the total distance driven in London divided by the number of cars. Data on total distance driven in London is sourced from the Department for Transport’s road traffic statistics^49^. Each car is assumed to drive the same annual distance. Depending on the modal shift implemented, the future annual distance driven in London can decrease by a certain percentage over a set period time, specified as a policy input. For the modal shifts explored in the paper, changes in distance driven happen on shorter timescales, such as 5–10 years, than changes in the number of cars. This is because behavioural studies have shown that people find it easier to swap a journey to a different mode than to give up a car entirely^76^. Journeys by cars need to reduce before it’s at a low enough level for people to take the plunge and go car-free. If the baseline case is assumed, the distance driven increases from the 2019 value by 20% in 2050 following the trend from the Department for Transport data.

This study explores the best-case scenario of policies, such that the re-bound effect from increasing vehicle efficiency (such as through driving EVs or light-weighting) to increasing traffic is not included. In the UK, improving vehicle efficiency has resulted in increased travel with a rebound effect of around 5%^77^ which would reduce the effectiveness of the ‘improve’ policies further.

### Use-phase emissions and energy demand

The annual use-phase emissions of a car in a given year is dependent on the annual distance driven, *d*, the fuel consumption per unit distance of the car, *F*, and the emissions intensity per unit fuel consumed of the car, *em*. Total annual use-phase emissions, *EM*, is found by summing over all cars in the model, *c*,

4 $${{EM}}_{Y}=\mathop{\sum}\limits_{c}{d}_{Y}\times {F}_{c,Y}\times {{em}}_{f,Y}.$$

Similarly, annual use-phase energy demand is given by,

5 $${{EN}}_{Y}=\mathop{\sum}\limits_{c}{d}_{Y}\times {F}_{c,Y}\times {{en}}_{f,Y},$$

where *en* is the energy intensity of the fuel consumed.

For ICEVs, fuel consumption statistics for the average new car in the UK in litres of petrol or diesel consumed per 100 km is sourced from the Department for Transport^78^. This is multiplied by the emissions or energy intensities of fuels given by UK government conversion factors^79,80^. Average biofuel blend for petrol and diesel is assumed which is the typical blend when purchasing forecourt fuel in the UK. For petrol, this is 2.14805 kgCO_2_/litre and for diesel, 2.52058 kgCO_2_/litre from the 2022 conversion factors^79^. The future fuel consumption of new cars is assumed to stay the same as 2019 levels in the baseline case, but in light-weighting cases, it is assumed to decrease by 0.28 l/100 kg per 100 km journey^81^.

For EVs, electricity consumption is estimated at 15kWh/100 km by averaging the manufacturer’s reported electricity consumption per 100 km values for the three most common EVs in the UK in 2019; the Nissan Leaf, Tesla Model 3 and BMW i3^82^. Light-weighting BEVs is assumed to decrease at 1 kWh/100 kg per 100 km journey^81^. Plug-in hybrid cars are assumed to drive 39% in electric mode and 61% petrol mode, as found as the real-world utility factor by the ICCT^83^.

The emissions and energy intensity of electricity is estimated by first assuming a pathway from the current UK electricity mix to a 100% renewable electricity mix by a ‘net-zero’ date. To calculate the emissions intensity, the mix of energy sources that year is multiplied by the respective life-cycle emissions intensities of the energy sources. Additional emissions intensities are added such as electricity transmission and distribution infrastructure and energy storage facilities for renewable energy in the grid. Lithium-ion battery storage facilities were chosen due to the assumed short-term nature of storage needed. The electricity emissions intensity in a given year, ${{em}}_{{elec},Y}$, in gCO_2_/kWh is given by,

6 $${{em}}_{{elec},Y}=\mathop{\sum}\limits_{s}{P}_{s}\times {{LCA}}_{s}+{T}_{m}+{P}_{{lib}}\times {{LCA}}_{{lib}},$$

where *P*_*s*_ is the proportion of electricity supplied by energy source *s*, *LCA*_*s*_ is the life-cycle emissions intensity of energy source *s*, *T*_*m*_ is the emissions intensity of electricity transmission infrastructure, *P*_*lib*_ is the proportion of electricity passing through storage and *LCA*_*lib*_ is the life-cycle emissions intensity of battery-storage facilities.

To calculate the energy intensity, it is required to divide by the average efficiency of the electrical grid to account for energies lost in the thermal conversions of some fuels or in storage facilities for renewable energy. The average efficiency is calculated by multiplying the energy mix of electricity that year by the efficiencies of each energy source. A factor of 3.6 is included for converting kWh to MJ. The embedded energy of energy sources is included by dividing by the average EROI (energy return on investment) of the grid and adding this on. Similarly, the embedded energy of energy storage facilities is calculated by multiplying the proportion of energy that year coming from renewables, *P*_*ren*_, by the assumed storage needed for a 100% renewable electricity scenario, *P*_*storage*_, assumed to be 15% from National Grid Scenarios^84^. This is divided by efficiency of renewable energy sources (which includes the efficiency of the storage system), *eff*_*ren*_, and the EROI of the storage facility, *EROI*_*lib*_. The energy intensity of transmission and distribution infrastructure is included. Thus, the energy intensity of electricity in a given year, ${{en}}_{{elec},Y}$, in MJ/kWh is given by,

7 $${{en}}_{{elec},Y}=\frac{3.6}{\mathop{\sum}\limits_{s}{P}_{s}\times {{eff}}_{s}}\left(1+\frac{1}{{EROI}}\right)+{T}_{n}+\frac{{3.6\times {P}_{{storage}}\times P}_{{ren}}}{{{eff}}_{{ren}}\times {{EROI}}_{{lib}}},$$

where *eff*_*s*_ is the efficiency of the energy source, *EROI* is the average EROI of the energy mix that year given by ${EROI}={\sum }_{s}{P}_{s}\times {{EROI}}_{s},$ and *T*_*n*_ is the energy intensity of electricity transmission infrastructure. The pathway assumed and numerical values used are given in Supplementary Note 12.

This study assumes that the energy efficiency of the renewables and energy storage system is higher than fossil fuels, and the detail behind this assumption is explained in Supplementary Note 3.

### Embedded emissions and energy demand

Emissions and energy demand from the resource extraction, manufacture, maintenance, and end-of-life of vehicles are included and listed for each fuel type in Supplementary Note 13. Embedded emissions and energy demands are introduced when the car enters the model fleet. The proportion of embedded emissions in car manufacturing that comes from electricity, 22%^85^, follows the decarbonisation of the grid in the regulated manufacturing case. Emissions from building EV charging points are included in the EV embedded emissions. Emissions from constructing and maintaining roads are found by multiplying the emissions and energy intensities of asphalt by the area of road in London. All the numbers used are given in Supplementary Note 13.

### Well-to-tank emissions and energy demand

Well-to-tank (WTT) emissions and energy expenditure for petrol and diesel fuel are included in the well-to-tank category. WTT emissions are calculated by multiplying fuel consumption by a WTT emission factor given by government conversion factors^79^. WTT energy expenditure is calculated by multiplying the energy consumption of petrol and fuel by a WTT factor in MJ/MJ_final fuel_ from the JEC Well-to-Wheels report^86^.

### Modal shift magnitudes and emissions

Most municipal regions choose to set targets on the basis of the numbers of trips done by different modes rather than distances. Given that the amount of tailpipe emissions is proportional to distance travelled and not number of trips, it is hard to calculate whether such targets are sufficient. In order to account for this, the National Travel Survey (2019)^51^ was used. The dataset was restricted to only trips that both began and ended in the Greater London Region. A random selection of trips was then selected in line with the number of trips being expected to be modally shifted in order to calculate how much of the total vehicle mileage was avoided this way. This was repeated 2000 different times and averaged in order to determine how many vehicle-miles would be displaced under different trip modal shift rates. It should be noted that limiting to trips beginning and ending in Greater London limits our study to ignore longer trips which make up disproportionately large contributions to emissions. However, it is unclear how to categorise them to London and the policies being discussed were those that would apply within the GLA region.

To calculate replacement mode emissions, the difference between distance driven in the baseline case, which assumes a 20% increase in 2050 from 2019, and distance driven in the modally shifted case, is calculated. This distance is multiplied by an average emissions intensity in gCO_2_/km of non-car modes. This intensity is calculated by using the modal share for London in 2019, normalising without the car mode, and multiplying the proportion of modes of a given journey, e.g., walk, cycle, rail and bus, by the appropriate emissions intensity of that mode. The average London occupancy for rail and busses is assumed, taken from UK Government reporting. The emissions intensities include embedded emissions from infrastructure, vehicles and active travel equipment^55^. Busses are assumed to instantly convert to electrical busses and decarbonise at the pace of the electrical grid decarbonisation assumed in other parts of the model. The values used are given in Supplementary Note 14.

The percentage of travel by each mode and public transport occupancies are assumed to stay constant at London’s current levels and busses are assumed to be electric with emissions decarbonising at the rate of the electrical grid assumed in the model.

### Carbon budget

The carbon budget considered in this study is directly comparable to tailpipe emissions only which occur within the boundary of London. This is due to the territorial-based carbon accounting methods used by the Kyoto Protocol which assigns emissions to their place of production. Since embedded emissions and electricity generation emissions usually occur outside of a local area, e.g., London in our study, the local carbon budget does not take them into account. Nevertheless, upstream and downstream carbon emissions should still be minimised and there is increased discussion around how best to account for these^87^.

We include the CCC’s 1.5 °C compatible pathway for surface transport as it is the current 1.5°C scenario used for governmental policymaking, and we apply grandfathering principles to obtain the relevant proportion for cars in London which results in cumulative emissions of 49 MtCO2eq. In addition, we include a more stringent estimate of the carbon budget for London from the Tyndall Centre^56^ due to its increased fairness approach, as it removes necessary global cement production and allows non “developed” nations to increase emissions to 2025 (see Supplementary Note 2 for more details). The carbon budget from a local analysis of London by Element Energy is included too. We also consider annual percentage reductions including the global 1.5 °C IPCC target of 45% emissions reduction by 2030 and net-zero in 2050^9^ and the 1.5 °C lifestyles target which represents the UK emission reductions required for equal per capita global emissions in 2030^22^.

The Tyndall carbon budget is estimated by taking the Tyndall Centre’s carbon budget for the London area which follows the “well below 2 °C and pursuing 1.5 °C” global temperature target and equity principles in the United Nations Paris Agreement^46^. The carbon budget for London is 260.9 MtCO_2_ for 2018–2100 and London’s emissions from 2018–2019 at 64 MtCO_2_ have been subtracted^47^. The remaining carbon budget is then multiplied by the percentage of London’s emissions which currently come from cars, 11%^47^.

Methodology behind the other carbon budgets used is given in Supplementary Note 1.

### Limitations of the model

The most important limitations of the model are listed here and further limitations in Supplementary Note 15.

#### Limited scope of city-level

At a national level, there would need to be a greater consideration of other modes that are available to rural areas, including special consideration of the least population dense areas of the UK where driving may be the only possible means. Studying average trips made and which trips can be modally shifted in rural areas would be beneficial.

#### Limited scope of cars

Decarbonisation pathways of other modes are not considered, including heavy-goods vehicles (HGVs) which may be harder to decarbonise due to technological limitations. Thus, a greater portion of the carbon budget may need to be assigned to other areas of surface transportation.

#### Carbon budgets

Carbon budgets examined do not take into account emissions occurring outside the boundary of London such as embedded emissions. This may present another limitation to the types of policies available in the transition to sustainable mobility. Carbon budgets in CO_2_ are not directly comparable to emissions results in CO_2eq_, so a slight discrepancy needs to be accounted for where these are compared.

#### Car travel activity

Car fleet size is defined exogenously so any dependency between other policy parameters and fleet size is not accounted for. In addition, this study does not explore which policies quantitively affect car travel activity.

## Supplementary information

Supplementary Information

## Data Availability

The data generated in this study and used in Figs. 1, 3 and 4 and Supplementary Figures are provided in the Source Data folder. Source data are provided with this paper.

## Source data

Source Data

## Code availability

The code can be found at https://github.com/LisaOJWinkler/UTPM.
