# Supplementary material for: The effect of sustainable mobility transition policies on cumulative urban transport emissions and energy demand
Source: Nat Commun. 2023 Apr 24;14:2357. doi: 10.1038/s41467-023-37728-x (PMC10125996; doi:10.1038/s41467-023-37728-x)
Supplement: Supplementary file 1 — Supplementary Information [file 41467_2023_37728_MOESM1_ESM.pdf]

Supplementary Information

The effect of sustainable mobility transition policies on cumulative urban transport emissions and energy demand

Lisa Winkler[1*], Drew Pearce[1], Jenny Nelson[1,2], Oytun Babacan[2,3*]

[1]Department of Physics, Imperial College London, London, UK. [2]Grantham Institute – Climate Change and the Environment, Imperial College London, London, UK. [3]Transdisciplinary Studies in Global Change, Brunel University, London, UK. [*]e-mail: lisa.winkler17@alumni.imperial.ac.uk, oytun.babacan@brunel.ac.uk.

Supplementary Note 1: Carbon Budgets

Global carbon budgets specify the remaining cumulative emissions that can be emitted for a percentage chance of remaining under a certain global temperature rise. From the IPCC Sixth Assessment Report (WG1), the 1.5°C and 2°C carbon budgets are as follows:

- Carbon budget of 500 $GtCO_2$ represents 50% chance of limiting warming to 1.5°C.
- Carbon budget of 900 $GtCO_2$ represents 17% chance of limiting warming to 1.5°C and 83% chance to 2°C.
- Carbon budget of 1350 $GtCO_2$ represents 50% chance of limiting warming to 2°C.

These global carbon budgets are inherently uncertain due to [1]:

- The sensitivity of the climate to GHG emissions and to what probability global warming will be kept below a certain temperature level
- Definitions of global warming e.g. 1.5 or 2 degree temperature rise relative to pre-industrial levels (1850-1900)
- Whether non-CO_2 emissions are accounted for as well as other climate forcers such as aerosols
- Whether an overshoot is assumed which will be removed with future carbon removal technologies

These factors can individually alter the carbon budget of a 50% chance of meeting 1.5°C between approximately -50% and +80% [1]. In addition to this uncertainty, there exists additional uncertainty in how the global carbon budget is allocated to individual countries, as well regions and economic sectors in that country. It is not as straight-forward as allocating the carbon budget equally by population, as many developed countries have had a significantly higher share of historic emissions. Thus, some argue that developing countries should be allocated a higher future share of emissions to aid development progress [2]. Others argue that developed countries have a greater reliance on fossil fuels, thus increasing the challenge of decarbonisation, especially as much of the built infrastructure is dependent on fossil fuels, and so should be allocated more of the global budget. Although the Paris Agreement does not allocate the global carbon budget to individual countries, it defines a set of criteria on how fairness should be achieved. It requires the 'highest possible ambition' from all countries, and that countries put forward commitments that reflect their common-but-differentiated responsibilities and respective capabilities [3]. However, this definition is vague, and there has been much discussion on what defines a 'fair' carbon budget in the literature [4]. A detailed analysis of fairness and carbon budget allocation is out of the scope of this study. Rather, we take various carbon budgets from governmental sources, policy think-tanks and scientific literature, and allocate these to the share for car transport in London by a proportion based on historic shares of emissions or car distance travelled. These budgets are listed in Supplementary Table 1 and the details on how national or surface transport budgets are allocated to London cars is explained below.

<u>Grandfathering by emissions</u>

London carbon budgets, such as the Tyndall centre budget for London, are allocated to car transport by multiplying the budget by the percentage of London's emissions which currently come from cars, 11% [5]. This is referred to allocation by historic emissions.

<u>Grandfathering by car use</u>

National carbon budgets are allocated to London using the percentage of total distance driven by cars in London over the UK. Due to the boundaries of the model, we are not allocating the budget by London's population over the UK; only the distance driven in London is included in the model, and the distance driven by Londoners elsewhere in the UK is not included. Therefore, the model boundary is territorial (London borders) rather than consumption (London population). In addition, in the case

of transportation, urban environments with their dense populations require less distance to be driven between people and amenities and so car use as well as the length of public transport infrastructure is smaller, and so the carbon budget should reflect these shorter distances. Taking the Department for Transport's (DfT) values of kilometres driven nationally in GB versus in London, the national value is 6,673 vehicle-km per capita for 2020 whilst London is 3,100 vehicle-km [6]. Assuming this proportion of distance driven, the carbon budget allocation to London is just 28.1/447.8 = 6%.

Grandfathering by emissions

Surface transport carbon budgets can be allocated to cars by assuming the percentage of surface transport emissions in the UK which currently come from cars. From the CCC's 6th carbon budget report [1]: "Total emissions from surface transport in 2019 were 113 $MtCO_{2eq}$, comprising 22% of total UK GHG emissions. These are primarily tailpipe emissions from fossil-fuelled road vehicles, with cars (68 MtCO2e) the largest contributing types." Therefore, cars represent 60% of surface transport emissions.

Allocation by population

The budgets taken from the CCC 6th carbon budget report are given in tCO_2/person, so we multiply by population of London to estimate the carbon budget for London. Then, a grandfathering by emissions approach is used to allocate this to cars.

Supplementary Table 1: Carbon budgets and their assumptions and emissions allocation approach.

**Source**	**London Carbon budget**	**Resulting Budget for Cars**	**Warming**	**Assumptions and Allocation Approach**	**Name of budget in figure**
Tyndall [7]	260.9 MtCO2 (2018-2100)	21.7 MtCO2	17% chance of limiting warming to 1.5 °C and 83% chance to 2 °C	The carbon budget for London is 260.9 $MtCO_2$ for 2018-2100 and London's emissions from 2018-2019 at 64 MtCO2 have been subtracted. The remaining carbon budget is then multiplied by the percentage of London's emissions which currently come from cars, 11% (**grandfathering by emissions**).<br>Tyndall remove from the total budget 60Gt due to necessary cement production and allow non "developed" nations to increase emissions to 2025 before the remaining budget is allocated. Tyndall then further deduct aviation and shipping emissions saying that's beyond the realm of a local councils authority before dividing by population.	Tyndall carbon budget in Fig 1 and Fig 4
IPCC AR6WG 1 [8]	580 MtCO2	63.8 MtCO2	50% chance of limiting warming to 1.5 °C	Global budget distributed equally to London's **population**. Then multiplied by 11% for cars – **grandfathering by emissions**	N/A
IPCC AR6WG 1 [8]	1040 MtCO2	114.4 MtCO2	17% chance of limiting warming to 1.5 °C and 83% chance to 2 °C	Global budget distributed equally to London's **population**. Then multiplied by 11% for cars – **grandfathering by emissions**.	N/A
CCC 6th Carbon Budget		49 MtCO2e	Compliant with Paris Agreement	We take the CCC 6th Carbon Budget's Balanced Net Zero Pathway for surface transport which is compliant with the Paris Agreement as a budget. For surface transport, the cumulative emissions between 2020-2050	CCC 1.5°C Pathway in Fig 1

report [1]				are 1356 $MtCO_{2eq}$. We multiply this by 60% for cars and by 6% for distance driven in London to get 49 $MtCO_{2eq}$. **Grandfathering by emissions and car use**<br><br>CCC assume UK has above global average duty to reduce emissions, but is also constrained by practical emissions reduction potential	
CCC 6th Carbon Budget report [1]	575 MtCO2 (2020-2050)	63.25 MtCO2	UK – Balanced Net Zero Pathway	Cumulative CO2 emissions (2020 – 2050)<br><br>[tCO2/person] from CCC 6th Carbon Budget Report (Table 7.1) multiplied by population of London, then multiplied by 11% for cars. Allocation by **population and grandfathering by emissions**<br><br>CCC assume UK has above global average duty to reduce emissions, but is also constrained by practical emissions reduction potential	CCC Balanced Pathway in Fig 4
CCC 6th Carbon Budget report [1]	422 MtCO2 (2020-2050)	46.42 MtCO2	~50% 1.5°C	Cumulative CO2 emissions (2020 – 2050)<br><br>[tCO2/person] from CCC 6th Carbon Budget Report (Table 7.1) multiplied by population of London, then multiplied by 11% for cars. Allocation by **population and grandfathering by emissions**<br><br>CCC assume UK has above global average duty to reduce emissions, but is also constrained by practical emissions reduction potential	CCC Global Avg. ~50% 1.5°C<br><br>In Fig 4
CCC 6th Carbon Budget report [1]	655 MtCO2 (2020-2050)	72.05 MtCO2	>66% 2°C	Cumulative CO2 emissions (2020 – 2050)<br><br>[tCO2/person] from CCC 6th Carbon Budget Report (Table 7.1), multiplied by population of London, then multiplied by 11% for cars. Allocation by **population and grandfathering by emissions**<br><br>CCC assume UK has above global average duty to reduce emissions, but is also constrained by practical emissions reduction potential	CCC Global Avg. >66% 2°C<br><br>In Fig 4
Element energy [9]	289 $MtCO_{2eq}$	32$MtCO_{2eq}$	Accelerated Green - 1.5°C Compatible	Multiply the emissions pathway for London for the Accelerated Green scenario by 11% for cars. **Grandfathering by emissions**	Element Energy Accelerate d Green in Fig 4
Climate action tracker [10]	832 MtCO2e (2020-2100)	91.52 MtCO2	1.5°C	Modelled pathway for 1.5C global emissions summed across 2020-2100 740GtCO2e and scaled down by population to London to 832MtCO2e (Kyoto basket)	N/A
Fair Share from Climate	Negative budget	0 MtCO2	1.5°C	Uses 40+ sources on fair emissions budgets to establish the UK as having a negative fair share emissions allowance, meaning that by 2030, the government would have to have	N/A

Action Tracker [10]				fully phased out its emissions or compensate its remaining emissions with reductions elsewhere, for example through supporting emissions reductions in other countries	

Supplementary Note 2: Modal Share to KM Stochastic Model

Many sustainability goals set by municipal authorities will target a maximum particular modal share of all trips being completed by private car, for example the Mayor of London's target for London being a 20% private car modal share by 2041 [11]. In order to estimate the emissions reduction associated with this, it is imperative to convert the modal share percentage to car distance driven in kilometres because CO_2 emissions are proportional to the distance travelled rather than the share of trips. Hence, we use the National Travel Survey 2019 [12] for car trips originating in London, which represents the current modal share. For a given target modal share for private cars, the corresponding number of trips are selected at random and the number of kilometres is summed. This is repeated a thousand times and averaged. Thus, our stochastic model is assuming that the length of the trip has no effect on the likelihood of it being modally shifted.

Supplementary Note 3: Energy Demand of Electricity Decarbonisation

This study assumes that the energy efficiency of the renewables and energy storage system is higher than fossil fuels, however there may be cases where renewables and storage are more energy intensive than relying on incumbent fossil fuel options. Although renewable energy sources such as wind and solar have usually smaller, and in some cases similar, energy return on investment (EROI) to fossil fuel generation, the volatility of renewables may require deploying large amounts of energy storage that would reduce overall EROI of the system especially if demand and supply systems are not well coordinated.

Supplementary Note 4:

Supplementary Figure 1: Sensitivity of cumulative tailpipe emissions to the magnitude and rate of car travel activity reduction for the combined policy case which assumes BEVs to be powered by 100% renewable electricity, a 2025 fossil fuel phase-out, 33% retrofitting, 40% light-weighting, strict standards on EV manufacture. Applying a reduction in car travel activity ranging from -50% to -90% by 2027 results in a cumulative tailpipe emissions range of 8 $MtCO_{2eq}$. Exploring the second dimension of rate of car travel activity also represents a relatively low amount of carbon for very differing efforts of carbon mitigation. Changing whether a 50% reduction in CTA is achieved in 3 years rather than 13 years from 2022 results in a cumulative tailpipe emissions savings of 6.5 $MtCO_{2eq}$.

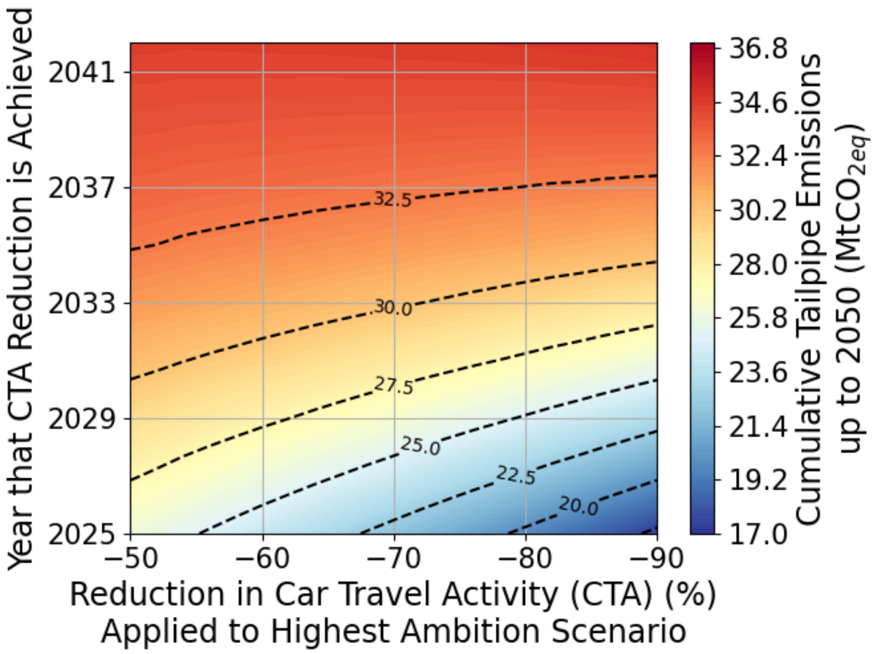

Supplementary Note 5:

Supplementary Figure 2: The relationship between cumulative CO_2 emissions and energy demand for different policy combinations. Sum of use-phase and embedded emissions and energy demand are plotted for varying magnitudes of modal shift, scrapping, light-weighting, retrofitting, ICEV phase-out, regulated EV manufacture and electricity decarbonisation policies. Magnitudes of modal shift are distinguished by colour with purple corresponding to a modal shift of -80% and yellow to a modal shift of +20% (baseline case). The results are further divided by EV manufacture regulations, where vertical dashes correspond to model results where stricter emissions standards for the manufacture of EVs have been set and horizontal dashes correspond to no such emission standards set.

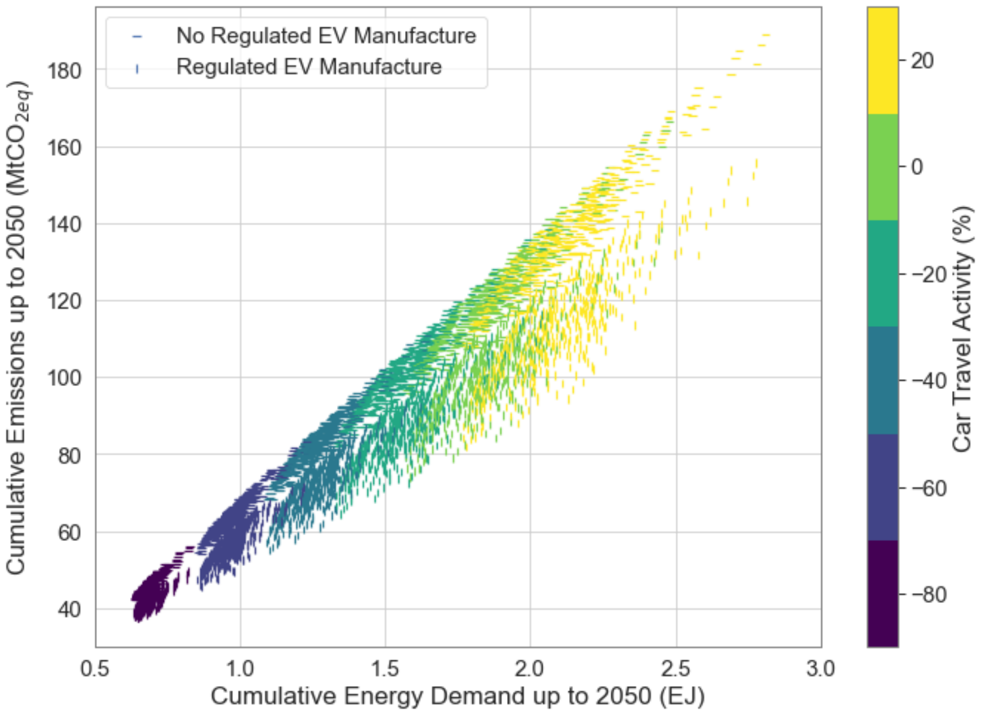

Supplementary Figure 2 shows the total cumulative emissions results against the total cumulative energy demand results of different combinations of policies including car travel activity reduction, scrapping, light-weighting, retrofitting, ICEV phase-out, regulated EV manufacture and electricity decarbonisation. The colour of the results corresponds to the reduction in distance driven, with purple corresponding to a reduction of -80% (darkest) and yellow corresponding to a baseline travel activity increase of +20% (lightest). Every incremental decrease in distance driven results in a clear decrease in emissions and energy demand. Thus, reducing car travel activity is the biggest driver of emissions and energy reduction.

In addition, emissions and energy demand are highly correlated such that policy combinations that result in the lowest emissions are also those that result in the lowest cumulative energy demand. Thus, even assuming low carbon electricity supply can always meet demand, reaching emissions targets requires reducing energy demand greatly.

Supplementary Figure 2 also demonstrates that decreasing car travel activity narrows the distribution of emissions and energy demand results. This is because at low levels of car travel activity, car-related policies become much less significant. Since policies such as light-weighting and ICEV phase-out only affect new cars, these policies would require a large influx of new cars to make a substantial impact on emissions. However, at low levels of car activity, the demand for new cars is greatly decreased, so the impact of these policies is also limited, creating less variation of emissions and energy demand results. Similarly, as the distance driven is greatly reduced, retrofitting and electricity decarbonisation policies which target the use phase emissions of cars have a limited impact.

The results in Supplementary Figure 2 are further subdivided by their EV manufacture regulation, where vertical dashed lines correspond to EV manufacture regulations and horizontal dashed lines to no EV manufacture regulations. These regulations refer to the strict emissions standards that can be set on the manufacture of the batteries and other components of an electric vehicle, such as powering factories by renewable energy rather than fossil fuels, as well as utilising less emissions intensive manufacturing processes. Such a decrease in emissions intensity of car manufacture leads to a decrease in total emissions results. It also shifts the correlation between energy and emissions down, thus being a highly favourable policy for targeting the emissions intensities of vehicle production.

Supplementary Figure 3 shows the probability densities of cumulative emissions results at varying magnitudes of car travel activity for 0% and 40% light-weighting. As also seen in Supplementary Figure 1, a low level of car activity narrows the distribution of emissions results because the impact of car-related policies become less significant. It also provides more certainty on emission reduction compared to the baseline (+20%) level of car travel activity which has a range of more than 100 $MtCO_{2eq}$ compared to the 20 $MtCO_{2eq}$ range seen for -80% car travel activity in Supplementary Figure 2. Thus, when car travel activity is reduced substantially, the contributions of other policies are dwarfed. Reducing car activity trumps all other emission mitigation efforts and allows for room to run behind on other policies. Therefore, funding and policy efforts should be placed first and foremost there.

Supplementary Figure 3: The probability distribution of cumulative emissions results with respect to modal shift and light-weighting. The sum of use-phase and embedded emissions from 2020 to 2050 for the London car fleet is shown as a probability density with respect to modal shift. On the right, the distribution is shown for no light-weighting and on the left for 40% light-weighting. The probability density is shown at four magnitudes of modal shift (-80%, -60%, -40%, and 0%) and the distribution gets narrower as modal shift intensifies because the impact of car-related policies become less significant. Note that the two peaks seen in the distributions arise from the two cases of regulated EV manufacture policies and no regulated EV manufacture policies.

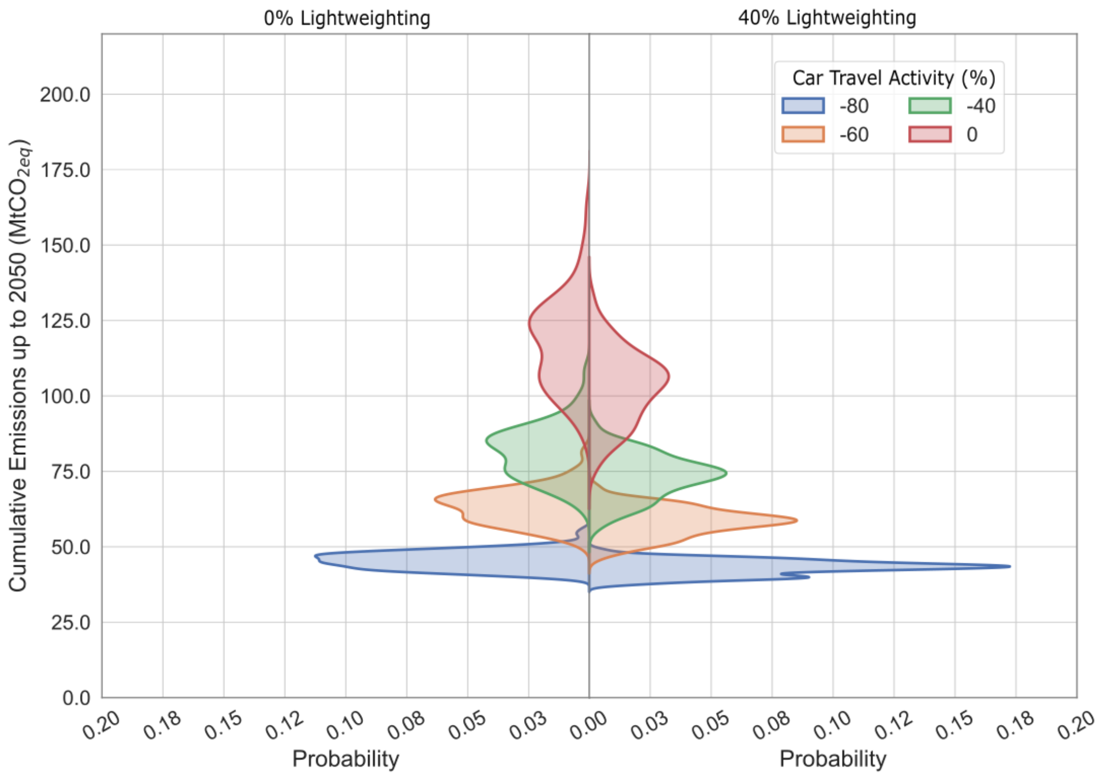

Light-weighting new cars results in a small shift of the emissions distributions down so that the minimum emissions result of the light-weighting scenario is slightly lower than no light-weighting. However, the biggest impact is in the shift of the probability peak down toward lower cumulative emissions. This means that light-weighting new cars results in a higher certainty of achieving an emissions reduction than no light-weighting at all. The policy also has the effect of narrowing the distribution. This is because when new cars are lighter and less emissions-intensive, there is less chance for emissions to be extremely high (for example in the case of an increase in car travel activity or a late phase-out) as light-weighting will reduce the emissions associated with those cases. Thus, the variability between emissions results of the baseline case and highly disruptive policies is minimised. In other words, light-weighting limits the run-away effect of 'ineffective climate' policies. Thus, combining policies which all work to reduce the emissions-intensities of cars localise emissions results and provide certainty on the cumulative emissions associated with these policies.

Supplementary Note 6:

Supplementary Figures 4 and 5 explore the emissions and energy impact of different combinations of policies including modal shift, scrapping, light-weighting, retrofitting, ICEV phase-out, and electricity decarbonisation. Emissions and energy demand closely follow the same relationships and trends in the figures. Thus, policy combinations that result in the lowest emissions are also those that result in the lowest cumulative energy demand.

The modal shift, light-weighting, retrofitting, ICEV phase-out and electricity decarbonisation policies are monotonic in the sense that a greater magnitude of the policy results in greater emission reductions. The scrapping policy, on the other hand, results in greater emissions with too high and too low a scrap age. This is because there exists a balance between use-phase and embedded emissions. Although scrapping cars earlier results in use-phase emissions savings it can be outweighed by an increase in emissions from building new EVs to replace functional ICEVs. The optimal scrap age

depends on the policies introduced. For example, with a late 2040 phase-out date and no retrofitting as shown in Figure 5, scrapping prematurely results in an increase in emissions. Scrapping only becomes a feasible policy when it results in a replacement of an ICEV for an EV or a modal shift. In a wider context, a policy may be detrimental to emissions in the context of all the other policies in place and for a different set of wider policies. Such interdependent relationships should be explored further when evaluating region-dependent policy options.

The light-weighting, retrofitting, ICEV phase-out and electricity decarbonisation policies all make positive contributions to emissions reductions. However, when high magnitudes of modal shifts are implemented, the contributions of other policies are dwarfed. In Supplementary Figure 1, for an 80% modal shift, the cumulative emissions results remain within a 6 $MtCO_2$ range no matter the combination of policies. This means that the modal shift policy trumps all other emission mitigation efforts. It also allows for room to run behind on other policies. Nevertheless, the best policy combination is considerable light-weighting, achieving ‘net-zero’ in 2040, retrofitting a large proportion of existing ICEVs and phasing out in 2025. However, considering that modal shift is responsible for the greatest proportion of emission reduction, funding and policy efforts should be placed first and foremost there.

Supplementary Figure 4: The effect of light weighting and electricity decarbonisation when combined with modal shift and scrap age on the cumulative CO_2 emissions and energy demand. Total cumulative emissions and energy demand results from 2020 to 2050 for the London car fleet are shown. Total refers to the sum of cumulative use-phase, embedded and modal shift emissions and energy demand. Different combinations of policies are explored; light-weighting, electricity decarbonisation, modal shift and scrap age. The colours correspond to the magnitude of cumulative emissions or energy demand, whilst the numbers in each box correspond to the difference in magnitude between the global minimum, shown in blue, and the respective box.

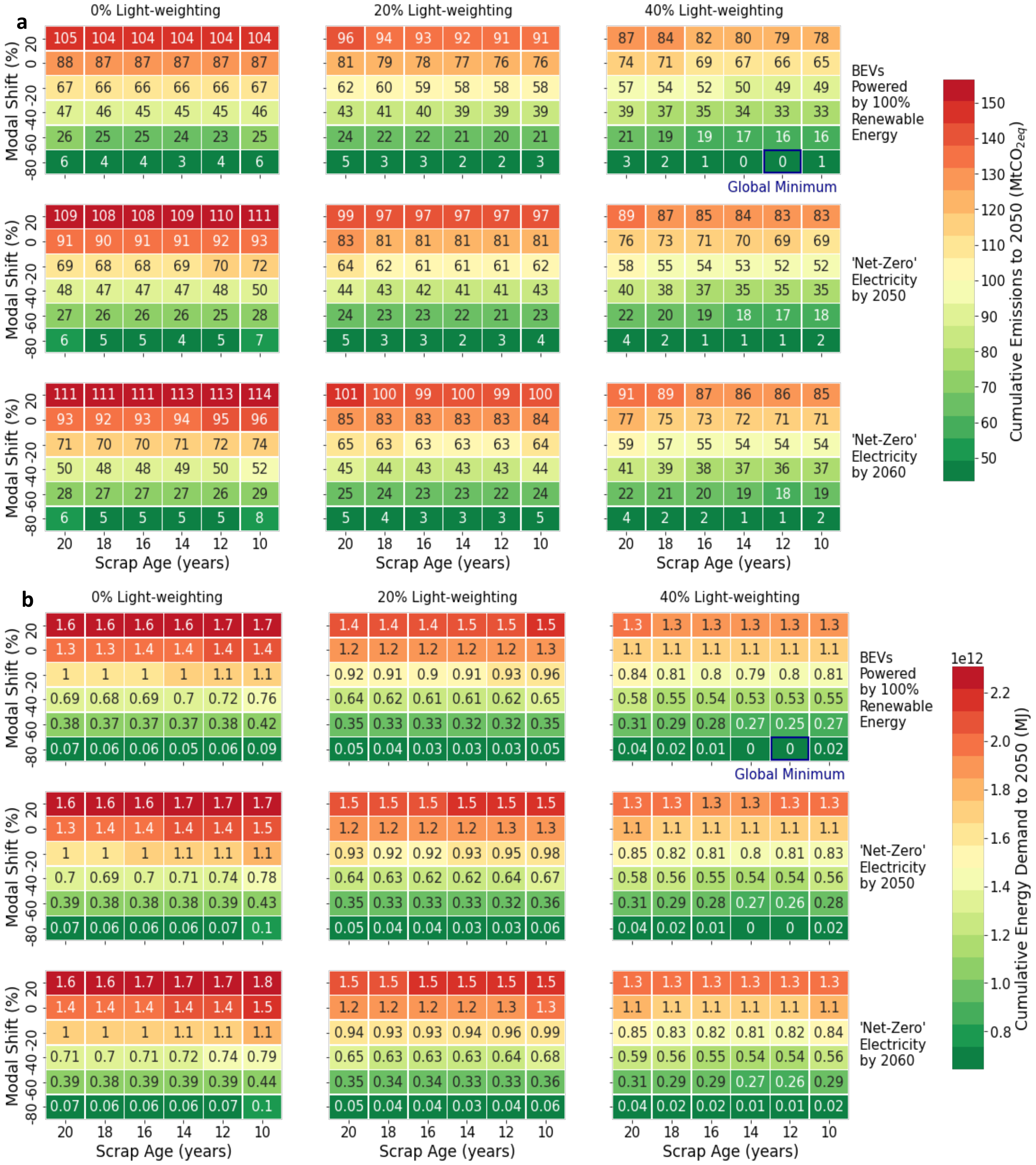

Supplementary Figure 5: The effect of retrofitting and fossil fuel phase-out when combined with modal shift and scrap age on the cumulative CO_2 emissions and energy demand. Total cumulative emissions and energy demand results from 2020 to 2050 for the London car fleet are shown. Total refers to the sum of cumulative use-phase, embedded and modal shift emissions and energy demand. Different combinations of policies are explored; retrofitting, phase-out, modal shift and scrap age. The colours correspond to the magnitude of cumulative emissions or energy demand, whilst the numbers in each box correspond to the relative difference in magnitude between the global minimum, shown in blue, and the respective box.

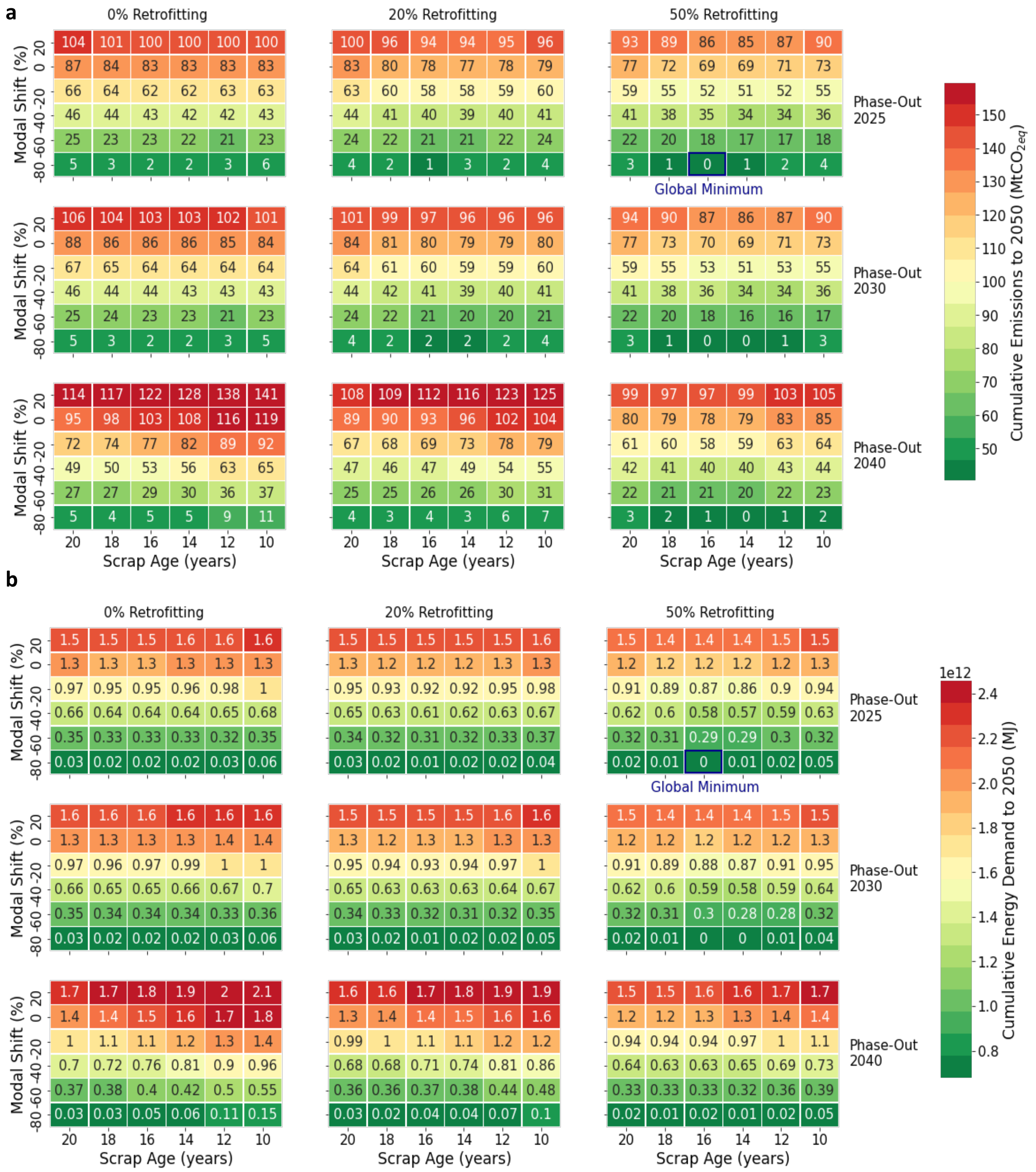

Supplementary Note 7: Limiting Number of Cars

Supplementary Figure 6 shows the results of the model with a modal shift of -81%, thereby reducing the number of cars in London by 81% by 2040, and two ICEV scrap ages of 10 and 15 years. The left panel shows the number of cars and the emissions results for the 10 year scrap age. The right panel shows the same outputs for the 15 year scrap age. Due to the younger age of scrappage, the number of BEVs sees a sharp increase and decrease around 2035 in subfigure a. This is due to the high adoption of BEVs and the remaining demand for cars that still exists at the time. However, once the demand diminishes in 2040, the BEVs are scrapped and removed from the fleet. This results in significantly higher embedded emissions, and greater emissions overall, as seen in subfigure c. If, however, a higher scrap age of 15 years is implemented, this behaviour is avoided as ICEVs are used for an additional few years until the demand for cars is much lower, which avoids the need to build entirely new vehicles. This shows that modal shift and scrap age policies need to be planned and forecasted simultaneously, as not to result in adverse emissions impacts from combining both policies together. To avoid the problem of building too many cars for a planned modal shift, policymakers and governments should focus on monitoring and limiting the number of cars each year in the future. The effect of wasted embedded BEV emissions can also be mitigated through retrofitting existing ICEV cars with electric engines to ease the transition from ICEVs to non-driving modes.

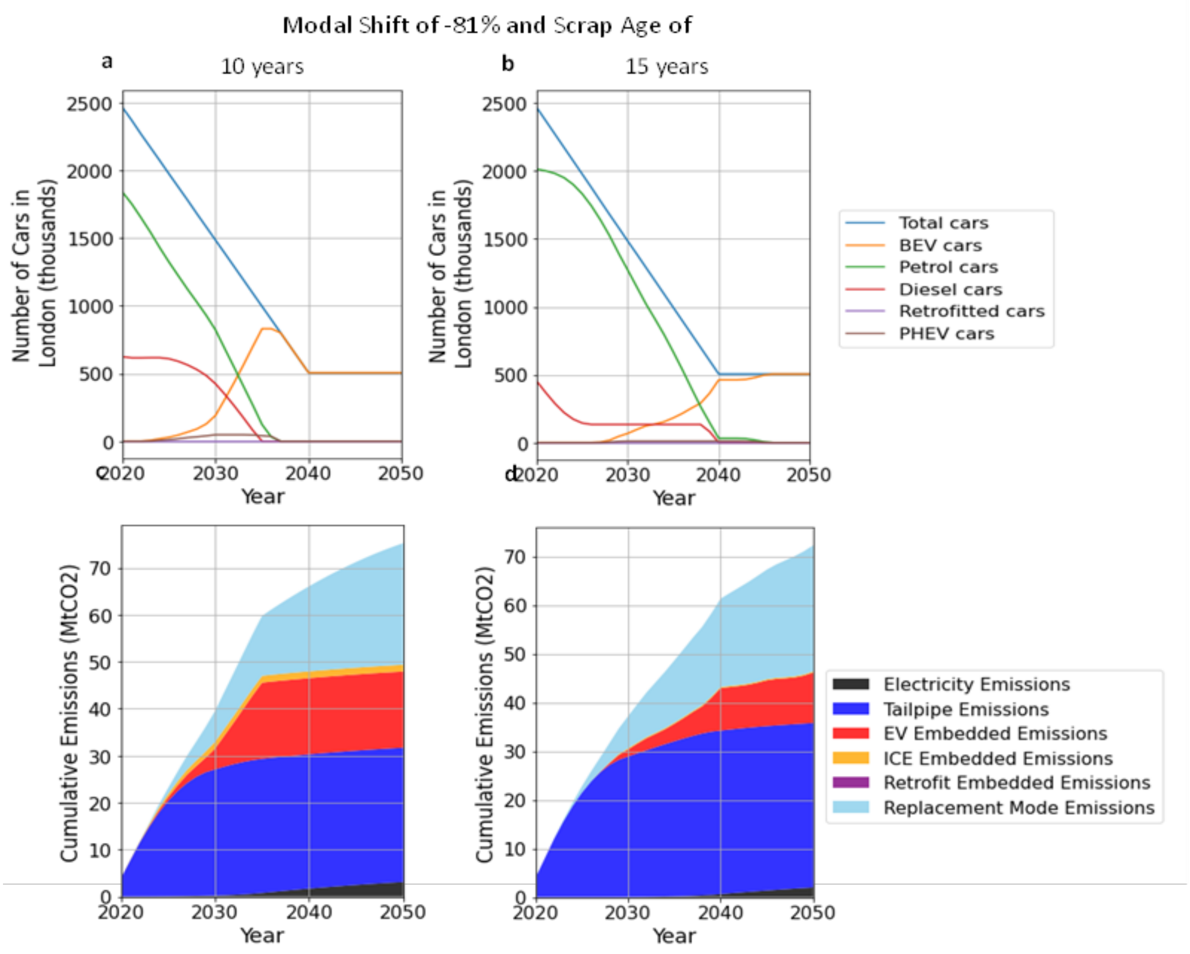

Supplementary Figure 6: Number of cars and cumulative emissions results for a modal shift of -81% and an ICEV scrap age of 10 years (left) and 15 years (right). Due to the dynamics of a modal shift and premature ICEV scrapping, less emissions are released when ICEVs are driven for longer, as this results in significantly fewer embedded emissions.

Supplementary Note 8:

Supplementary Figure 7: Relationship between energy demand and emissions for different rates of electricity decarbonisation. The contour lines and colours represent the cumulative emissions results for the model under different cumulative energy demand and electricity decarbonisation scenarios. Cumulative energy demand was changed by adjusting the levels of car travel activity. Electricity decarbonisation was changed by adjusting the year 100% renewable electricity generation is reached, referred to as ‘net-zero’. The later ‘net-zero’ is reached, the lower the energy demand needs to be to reach the same cumulative emissions.

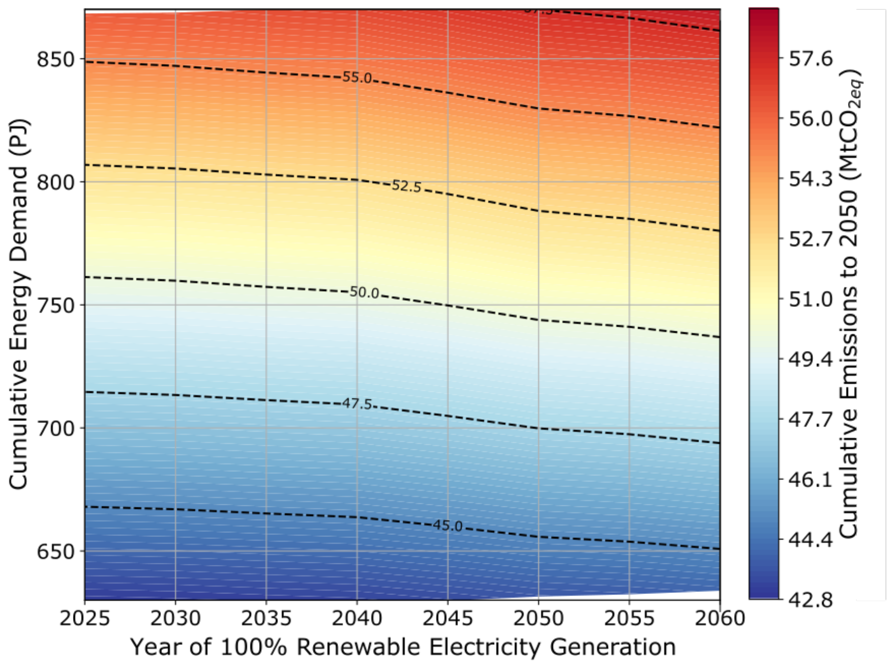

Supplementary Figure 7 shows the cumulative emissions associated with different rates of electricity decarbonisation and different cumulative energy demands, which was changed by adjusting the percentage of car travel activity. It shows that the same cumulative emissions can be achieved with slower rates of decarbonisation if energy demand is reduced. This is because the effectiveness of switching to electric vehicles as a carbon mitigation strategy is highly dependent on the emissions intensity of the electrical grid. Yet, the same cumulative emissions that can be achieved with ‘net-zero’ in 2025 can also be achieved with 3% less cumulative energy demand from cars and ‘net-zero’ in 2060. Emissions are far more sensitive to reductions in energy demand than to the emissions intensity of the grid. Thus, reducing energy demand from cars through modal shifts and policies such as light-weighting is a more effective carbon mitigation strategy than electricity decarbonisation for transport emission mitigation.

Supplementary Note 9: Car Fleet Initialisation

The Urban Transport Policy Model can be applied to different cities by adjusting the initialisation parameters - the values of inputs for car fleet initialisation listed in Supplementary Table 2 below. Using the model code available online, the inputs can be adjusted in a separate city-specific input file, separated from the main methods of the model.

Supplementary Table 2: List of inputs for car fleet initialisation. Adjusting these initialisation parameters allows the model to be applied to any city.

Input	Symbol and Value for London	Variable Name in Code	Source
Total number of cars in 2019	C – 2,661,000	cars_2019	Published regional data [13]
Age distribution in London's 2019 fleet	A – see source data	car_age	Published regional data [13]
Historic adoption rate (proportion of new cars sold of each fuel type)	P – see source data	adop_car_p, adop_car_d, adop_car_h, adop_car_ph, adop_car_b	Published regional data [13]
Average fuel consumption (litres per 100km) for new cars registered 1997-2019	F – see source data	fuel_car_p, fuel_car_d	Published regional data [14]
Distance driven per year by cars & taxis in London in 2019 (billion km)	D – 28.1 billion km	km_2019	Published regional data [6]

To initialise the fleet, the model creates car objects by following the procedure illustrated in Supplementary Figure 8. The number of car objects of a given age and fuel type is calculated by multiplying the car fleet size by the age distribution of the 2019 car fleet and the historic proportion of fuel types year-on-year (otherwise referred to as the historic adoption rate). The difference between the number of cars initialised by the model for London and data from the Department for Transport (DfT) is shown in Supplementary Table 3 below.

Supplementary Table 3: Difference between number of cars initialised by the fleet versus actual fleet size.

Car Type	London 2019 Fleet Size (DfT)	Number of Cars Initialised in Model	Difference	Total Difference (across all fuel types)
Petrol	1,618,000	1,604,000	-0.9%	-0.5%
Diesel	1,024,000	1,010,000	-1.4%	-0.5%
Plug-in Hybrid	13,000	10,000	-23%	-0.1%
Battery-Electric	8,000	6,000	-25%	<-0.1%
Total	2,661,000	2,630,000	-1.2%	-1.2%

Supplementary Figure 8: Flowchart of the car fleet initialisation process for the year 2019. By importing the 2019 fleet size, the age distribution of the fleet and the adoption rates of different fuel-types pre-2019, and looping over age and fuel type, a model car fleet is created comprising car objects with the attributes age and fuel type.

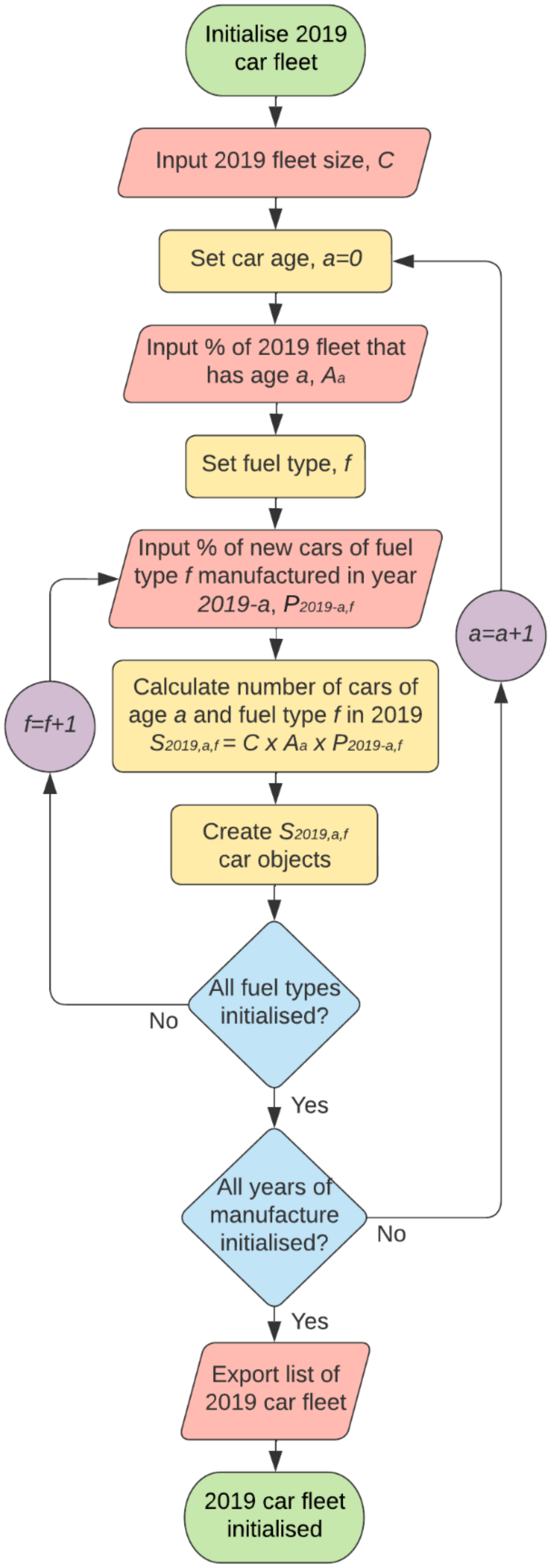

Supplementary Note 10: Fuel Adoption Rates

The adoption rates for all fuel types between 1989 to 2050 are shown in Supplementary Figure 8. Data for the adoption rates from 2001 to 2020 are sourced from the department for Transport [13]. Assumption made for post-2020 adoption rates are stated specifically in Supplementary Table 4.

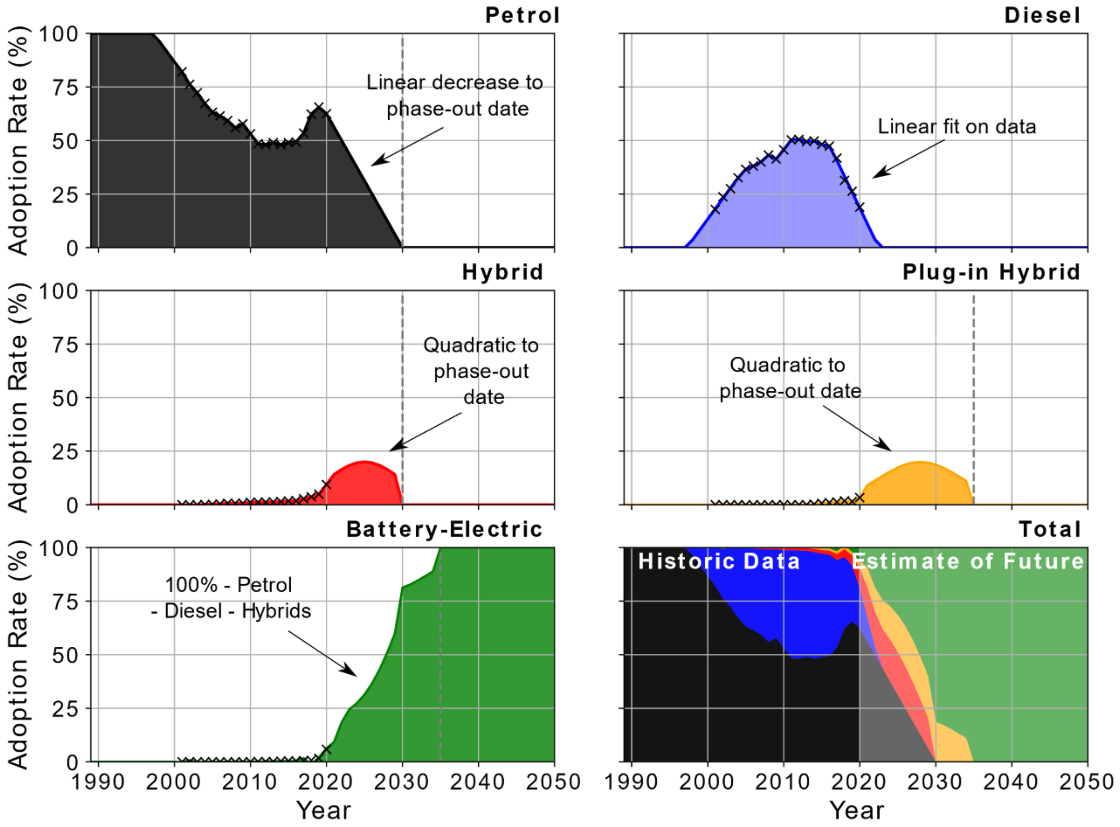

Supplementary Figure 9: Diagram of the adoption rates of each fuel type. Adoption rate is the percentage of new cars sold of a given fuel type. Petrol, hybrid and plug-in hybrid adoption is phased out at the phase-out date, which is a variable in the model. Diesel phases out to zero irrespective of the phase-out date. BEV adoption is the difference between 100% and other fuel types. Detailed assumptions are stated in Supplementary Table 4.

Supplementary Table 4: Assumptions on the future projection for each fuel type's adoption rate.

Fuel Type	Future Adoption Rate Assumptions
Petrol	Assumes a linear decrease from 2020 levels to 0% in the ICEV phase-out year.
Diesel	Assumes that diesel will decrease to 0% following the same rate of current decline of diesel adoption (from 2017 to 2020 data).
Hybrid	Following the diesel trend, the adoption of hybrids are assumed to increase to a peak and then decrease. Assumes a peak of 20% adoption halfway between 2020 and the ICEV phase-out date. Although hybrids are specified in the adoption rates, this category of cars are initialised under petrol cars in the model.
Plug-in Hybrids (PHEVs)	Similar to hybrids, assumes a peak of 20% halfway between 2020 and the plug-in hybrid phase-out date.
Battery-Electric (BEVs)	Taken to be the difference between 100% adoption rate and the sum of the adoption rates of petrol, diesel and both hybrid types. The battery-electric category represents all possible zero-emission vehicles.

Supplementary Note 11: Car Fleet Evolution

Policies are explored in the model by changing the value of policy parameters. These parameters are listed in Supplementary Table 5 below, specifying the policies which are explored by adjusting the parameter, the form of the variable, and whether these are directly modifiable by the user when running the model.

Supplementary Table 5: List of inputs for car fleet evolution. Adjusting these evolution parameters allows the model to undergo policy scenarios.

**Input**	**Policy**	**Form of variable**	**Modifiable by user?**
Future adoption rate	Electrification	%, per year, linear or quadratic function, dependent on phase-out year	No
Phase-out year (ban on sale of new ICEVs)	Electrification	Target year	Yes
Future fleet size	Car travel activity	% change over period base year – 2040, linear	Yes
Future distance driven – magnitude	Car travel activity	% change over period base year – target year, linear	Yes
Future distance driven – rate	Car travel activity	Number of years starting at 2022	Yes
Scrap age	Scrap and Replace	Cut-off age at which cars are scrapped	Yes
Electricity Grid Mix	Electricity Decarbonisation	% share, for each year, of coal, natural gas, solar, wind, nuclear, biomass, hydro, imports, dependent on year of net-zero	No
Year of net-zero (100% renewable generation)	Electricity Decarbonisation	Target year	Yes
Retrofit percentage	Retrofit	% of scrapped cars retrofitted	Yes
Average fuel consumption of fleet	Light-weighting	Litres/kWh per 100km, dependent on average car mass	No
Average car mass	Light-weighting	Mass relative to 1400kg	Yes

The model undergoes car fleet evolution upon importing the pre-initialised fleet and specifying the model parameters. Then, new and old cars are added and removed from the model every year. The number of new cars is defined as the difference between the size of the car fleet in that year and the size of the car fleet in the year before plus all old cars removed in that year due to age. The size of the car fleet for any given year is defined exogenously and follows a linear increase/decrease from the 2019 car fleet size to a specified fleet size in 2040, remaining constant thereon. The future adoption rate of the fleet for car fuel types is described in detail in Supplementary Note 10. The future distance

driven per year of the fleet is defined as a linear change from the 2019 distance driven in London to a target year in the future, e.g., a 20% reduction in distance driven by 2027.

Supplementary Figure 10: Flowchart of the car fleet evolution process including the emissions impacts.

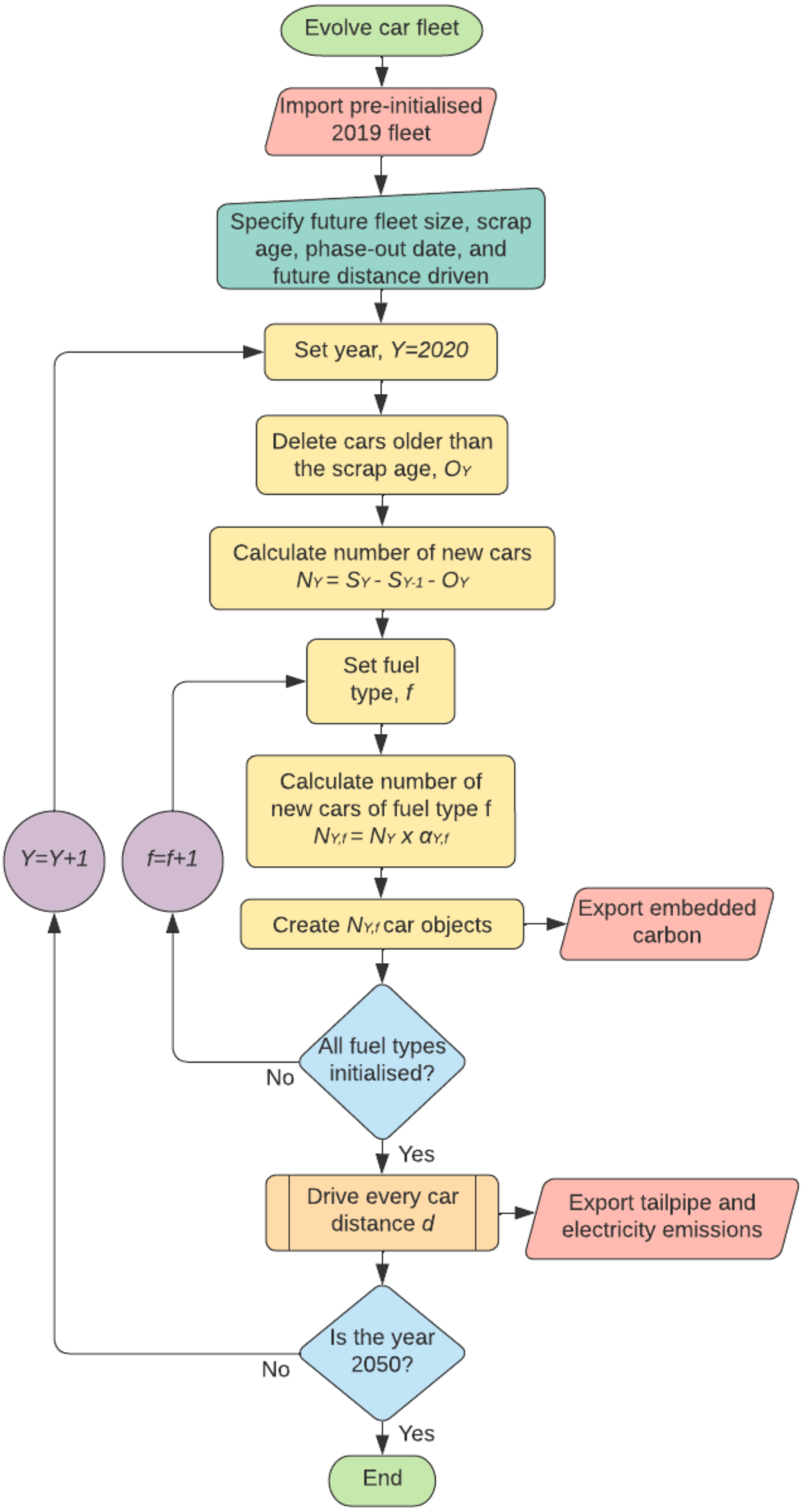

Supplementary Note 12: Electricity Mix and Emissions

Electricity mix assumptions are listed in Supplementary Table 6 and the diagram for the 100% renewable electricity generation in 2050 case is shown in Supplementary Figure 11. Forecast can be applied to reach 100% renewables at earlier or later dates by shifting the decadal electricity mix assumptions to the appropriate dates. The life-cycle emissions intensity, energy intensity (EROI or LCA analysis) and the efficiencies are listed in Supplementary Table 7.

Supplementary Figure 11: Proportion of energy sources in UK electricity generation reaching 100% renewable electricity generation in 2050. Decadal electricity mix values can be shifted to different dates to reach 'net-zero' earlier or later than 2050. Detailed assumptions used to make this forecast are listed in Supplementary Table 6.

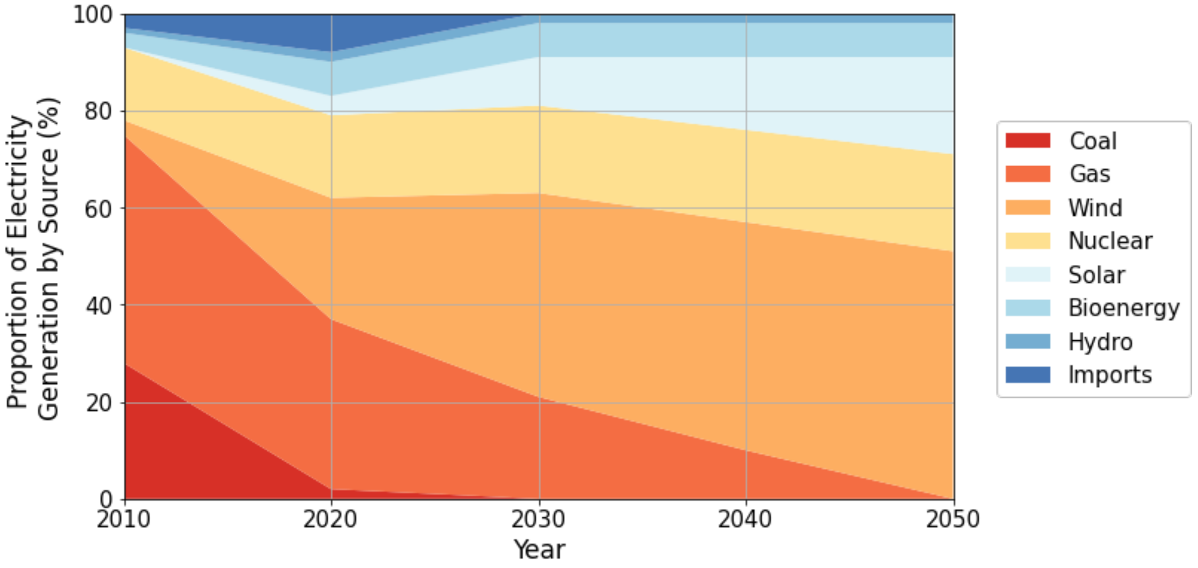

Supplementary Table 6: Detailed assumptions and corresponding references for the electricity mix forecast.

**Energy Source**	**Assumptions & References**
Historical Values	Electricity mix values are taken from the Department for Business and Industrial Strategy's electricity generation data [15]
Coal	Assume 0% coal electricity generation in 2030 and onward
Gas	Assume gradual decrease in gas use until zero in 2050. As most existing and new gas plants are Natural Gas Combined Cycle (NGCC), the emissions intensity for combined cycle is assumed [16]
Wind	The proportion of electricity generated by wind in 2030 was estimated from Boris Johnson's 10 point plan which aims to achieve a 40 GW wind power capacity by 2030, thus adding an extra 67% of 2020's wind power capacity [17]. The proportion from wind energy is assumed to increase to a maximum of 51% representing the primary source of UK energy.
Nuclear	Although Boris Johnson's 10 point plan is heavily backing nuclear power as an energy source [17], environmental concerns may slow down adoption past 20%, thus the proportion of electricity generated by nuclear fission is assumed to have a 1% decade-on-decade increase

Solar	In 2010 to 2020, the proportion of solar energy saw an increase of 5%, thus it is assumed that the proportion will increase by 5% every decade going forward [15]
Bioenergy	The proportion of bioenergy is assumed to stay constant at 2020 levels as increased burning of biomass is contentious in regard to land use and greenhouse gas emissions
Hydropower	Hydropower is also assumed to stay constant at 2020 levels as the UK's capacity for hydropower is almost maximised, with additional sites causing great environmental and conservation concerns [18]
Imports	For simplification, imported electricity is assumed to reach zero in 2030 and the life-cycle analysis value of it is taken as the average LCA value of all the other energy sources.

Supplementary Table 7: Values and references used for the energy source's emissions and energy intensities and efficiencies.

**Energy Source**	**Life-Cycle Emissions Intensity (gCO_2/kWh)**	**Ref.**	**EROI or Life-Cycle Energy Intensity**	**Ref.**	**Efficiency of System**	**Ref.**
Coal	980	[19]	46	[20]	32.1%	[16]
Gas	450	[21]	20	[20]	48.3%	[16]
Wind	10	[22]	20	[20]	100%	[16]
Nuclear	12	[23]	14	[20]	40.3%	[16]
Solar	45	[24]	10	[20]	100%	[16]
Bioenergy	29	[25]	5	[20]	34.7%	[16]
Hydro	31	[26]	84	[20]	99.6%	[16]
Transmission	11	[27]	190 kJ/kWh	[27]	/	/
Battery-Storage	43 for 1 cycle a day	[28]	4 for 1 cycle a day	[28]	82%	[28]

Supplementary Note 13: Embedded Emissions and Energy Demand

Supplementary Table 8: Embedded energies and emissions used in the model. Embedded vehicle values for the regulated manufacturing case are taken from the US study whilst the unregulated case are taken from the China study.

**Item**	**Process**	**Energy Demand**	**Ref.**	**Emissions**	**Ref.**
Vehicles	ICEV Production + EOL in US	69.7 GJ/vehicle	[29]	4235 $kgCO_2$/vehicle	[29]
	BEV Production +EOL in US	96.9 GJ/vehicle	[29]	8698 $kgCO_2$/vehicle	[29]
	PHEV Production + EOL in US	85.3 GJ/vehicle	[29]	5538 $kgCO_2$/vehicle	[29]
	BEV Production in China	93 GJ/vehicle	[30]	15000 $kgCO_2$/vehicle	[30]
	ICEV Production in China	63.5 GJ/vehicle	[30]	9985 kg $kgCO_2$/vehicle	[30]
	% Change with 10% Change in Mass	8.0% for ICEV<br>7.5% for BEV	[30]	7.3% for ICEV<br>6.7% for BEV	[30]

	Maintenance and repair	40 GJ/vehicle	[31]	3300 $kgCO_2$/vehicle	[31]
	ICEV to EV Conversion	Assume same proportion as BEV energy: 48.3 GJ/vehicle	[29] [32]	4340 $kgCO_2$/vehicle	[32]
Road	Asphalt road construction and maintenance	87.4 GJ/vehicle	[33] [34]	3447 $kgCO_2$/vehicle	[33] [34]
Electricity	EV Charging Points	1.36 MJ/kWh	[35]	94.06 gCO2/kWh	[35]
Fuel	Well-to-tank (fuel extraction, production and delivery 'to the tank')	Petrol – 0.23 $MJ/MJ_{final\ fuel}$ Diesel – 0.26 $MJ/MJ_{final\ fuel}$	[36]	Diesel (avg. biofuel blend) - 0.60986 $kgCO_{2eq}$/litre Petrol (avg. Biofuel blend) - 0.61328 $kgCO_{2eq}$/litre	[37]

Supplementary Note 14: Modal Shift Values

Supplementary Table 9: The 2019 values for the London non-car modal shares, calculated by normalising the modal shares without cars. The corresponding emissions intensity and energy intensity of the transport mode is given, where pkt corresponds to passenger kilometres travelled. For busses, the proportion of emissions from manufacture and infrastructure remains fixed, whilst the emissions from energy use decarbonises with the electricity grid.

**Mode**	**Non-Car Modal Share in London**	**Ref.**	**Emissions Intensity ($kgCO_2$/pkt)**	**Energy Intensity (MJ/pkt)**	**Ref.**
Walk	39%	[38]	0.00025	0.007	[39]
Cycle	4%	[38]	0.0094	0.1524	[39]
Bus	22%	[38]	0.1112	1.42	[39]
Rail	35%	[38]	0.0174	0.751	[39]

Supplementary Note 15: Model Limitations

Some of the limitations of the present analysis include:

- Limited scope of city-level: At a national level, there would need to be a greater consideration of other modes that are available to rural areas, including special consideration of the least population dense areas of the UK where driving may be the only possible means. Studying average trips made and which trips can be modally shifted in rural areas would be beneficial
- Limited scope of cars: Decarbonisation pathways of other modes are not considered, including heavy-goods vehicles (HGVs) which may be harder to decarbonise due to technological limitations. Thus, a greater portion of the carbon budget may need to be assigned to other areas of surface transportation
- Carbon budgets examined do not take into account emissions occurring outside the boundary of London such as embedded emissions. This may present another limitation to the types of policies available in the transition to sustainable mobility.
- Carbon budgets in CO_2 are not directly comparable to emissions results in CO_{2eq}, so a slight discrepancy needs to be accounted for where these are compared
- Magnitude of conclusions drawn from the study in terms of car travel activity are very sensitive to the carbon budget considered. For the combined policy case, a carbon budget of ± 4$MtCO_2$ results in an uncertainty in car travel activity of ± 20%

- No constraint in EV uptake is modelled, although in the real-world, EV uptake is constrained by EV availability

In the modelling of the policies:

- Decarbonisation of the grid: Only one decarbonisation pathway of the grid was analysed, although the rate of this decarbonisation was explored. Imported electricity was assumed to be zero
- Light-weighting: Follows a linear relationship with weight and is applied to the average fuel consumption per petrol and diesel fleet so assumes the entire new fleet is light-weighted rather than modelling a specific proportion of cars
- Regulated EV manufacture: Assumes that the energy demand of better manufacturing processes remains relatively similar, but that the carbon intensity of such processes decreases
- Retrofitting: Only the retrofitting of a percentage of scrapped cars is modelled which works to prolong the life of that proportion of cars. Large-scale retrofitting of entire fleets is not modelled due to the difficulty of implementing wide-scale retrofits on many car types at present. This could be analysed at greater scales in a future study to present an 'immediate' and 'extreme' policy scenario within retrofits
- Car travel activity: Car fleet size is defined exogenously so any dependency between other policy parameters and fleet size is not accounted for
- Modal shift model: Assumes bus fleet to instantly transition to electric in order to simplify model and avoid vehicle uptake modelling. Does not consider decarbonisation opportunities such as increasing occupancy of public transport and micro-mobility

References

[1] Comittee on Climate Change, "The Sixth Carbon Budget," 2020. [Online]. Available: https://www.theccc.org.uk/publication/sixth-carbon-budget/.

[2] H. Matthews, "Quantifying historical carbon and climate debts among nations," *Nature Climate Change,* vol. 6, pp. 60-64, 2016.

[3] United Nations Framework Convention on Climate Change, "The Paris Agreement," UNFCC, Paris, 2015.

[4] N. J. van den Berg, H. L. van Soest, A. F. Hof, M. G. J. den Elzen, D. P. van Vuuren, W. Chen, L. Drouet, J. Emmerling, S. Fujimori, N. Höhne, A. C. Kõberle, D. McCollum, R. Schaeffer, S. Shekhar, S. S. Vishwanathan, Z. Vrontisi and K. Blok, "Implications of various effort-sharing approaches for national carbon budgets and emission pathways," *Climatic Change,* vol. 162, no. 4, pp. 1805-1822, 2020.

[5] Greater London Authority, "London Energy and Greenhouse Gas Inventory (LEGGI)," GLA, London, 2021.

[6] Department for Transport, "Road traffic statistics," 28 09 2022. [Online]. Available: https://www.gov.uk/government/statistical-data-sets/road-traffic-statistics-tra.

[7] J. Kuriakose, C. Jones, K. Anderson, J. Broderick and C. McLachlan, "Setting Climagte Commitments for London: Quantifying the implications of the United Nations Paris Agreement for London," Tyndall Centre, Manchester, 2021.

[8] P. Arias, N. Bellouin, E. Coppola, R. G. Jones, G. Krinner, J. Marotzke, V. Naik, M. D. Palmer, G.-K. Plattner and J. Rogelj, "Climate Change 2021: The Physical Science Basis. Contribution of Working Group I to the Sixth Assessment Report of the Intergovernmental Panel on Climate Change," Cambridge University Press, Cambridge, United Kingdom and New York, NY, USA, 2021.

[9] Element Energy, "Analysis of a Net Zero 2030 Target for Greater London," Greater London Authority, London, 2022.

[10] Climate Action Tracker, "2100 Warming Projections: Emissions and expected warming based on pledges and current policies.," Climate Analytics and NewClimate Institute, November 2021. [Online]. Available: https://climateactiontracker.org/global/temperatures/.

[11] Greater London Authority, "Mayor's Transport Strategy," Mayor of London, London, 2018.

[12] Department for Transport, "National Travel Survey: 2019," 05 08 2020. [Online]. Available: https://www.gov.uk/government/statistics/national-travel-survey-2019.

[13] Department for Transport, "Vehicles statistics," 09 12 2020. [Online]. Available: https://www.gov.uk/government/collections/vehicles-statistics. [Accessed 23 04 2021].

[14] Department for Transport, "Energy and environment," 16 12 2020. [Online]. Available: https://www.gov.uk/government/statistical-data-sets/energy-and-environment-data-tables-env.

[15] Department for Business, Energy and Industrial Strategy, "Energy Trends: UK electricity (ET 5.1 - quarterly)," 30 09 2021. [Online]. Available: https://www.gov.uk/government/statistics/electricity-section-5-energy-trends. [Accessed 15 09 2021].

[16] Department for Business, Energy and Industrial Strategy, "Digest of UK Energy Statistics (DUKES): electricity," 29 07 2021. [Online]. Available: https://www.gov.uk/government/statistics/electricity-chapter-5-digest-of-united-kingdom-energy-statistics-dukes. [Accessed 05 09 2021].

[17] HM Government, "The Ten Point Plan for a Green Industrial Revolution," 11 2020. [Online]. Available: https://www.gov.uk/government/publications/the-ten-point-plan-for-a-green-industrial-revolution. [Accessed 16 04 2021].

[18] J. E. Sample, N. Duncan, M. Ferguson and S. Cooksley, "Scotland's hydropower: Current capacity, future potential and the possible impacts of climate change," *Renewable and Sustainable Energy Reviews,* vol. 52, pp. 111-122`, 2015.

[19] M. Whitaker, G. A. Heath, P. O'Donoughue and M. Vorum, "Life Cycle Greenhouse Gas Emissions of Coal-Fired Electricity Generation," *Journal of Industrial Ecology,* vol. 16, pp. S53-S72, 2012.

[20] C. A. S. Hall, J. G. Lambert and S. B. Balogh, "EROI of different fuels and the implications for society," *Energy Policy,* vol. 64, pp. 141-152, 2014.

[21] P. O'Donoughue, G. Heath, S. Dolan and M. Vorum, "Life Cycle Greenhouse Gas Emissions of Electricity Generated from Conventionally Produced Natural Gas," *Journal of Industrial Ecology,* vol. 18, pp. 125-144, 2014.

[22] S. Dolan and G. Heath, "Life Cycle Greenhouse Gas Emissions of Utility-Scale Wind Power," *Journal of Industrial Ecology,* vol. 16, pp. S136-S154, 2012.

[23] E. Warner and G. Heath, "Life Cycle Greenhouse Gas Emissions of Nuclear Electricity Generation," *Journal of Industrial Ecology,* vol. 16, pp. S73-S92, 2012.

[24] D. Hsu, P. O'Donoughue, V. Fthenakis, G. Heath, H. Kim, P. Sawyer, J.-K. Choi and D. Turney, "Life Cycle Greenhouse Gas Emissions of Crystalline Silicon Photovoltaic Electricity Generation," *Journal of Industrial Ecology,* vol. 16, pp. S122-S135, 2012.

[25] Committee on Climate Change, "Biomass in a low-carbon economy," 11 2018. [Online]. Available: https://www.theccc.org.uk/publication/biomass-in-a-low-carbon-economy/.

[26] H. L. Raadal, L. Gagnon, I. S. Modahl and O. J. Hanssen, "Life cycle greenhouse gas (GHG) emissions from the generation of wind and hydro power," *Renewable and Sustainable Energy Reviews,* vol. 15, no. 7, pp. 3417-3422, 2011.

[27] G. Harrison, M. J. Maclean, S. Karamanlis and L. F. Ochoa, "Life cycle assessment of the transmission network in Great Britain," *Energy Policy,* vol. 38, no. 7, pp. 3622-3631, 2010.

[28] T. Le Varlet, O. Schmidt, A. Gambhir, S. Few and I. Staffell, "Comparative life cycle assessment of lithium-ion battery chemistries for residential storage," *Journal of Energy Storage,* vol. 28, 2020.

[29] A. Mayyas, M. Omar, M. Hayajneh and A. R. Mayyas, "Vehicle's lightweight design vs. electrification from life cycle assessment perspective," *Journal of Cleaner Production,* vol. 167, pp. 687-701, 2017.

[30] Q. Qiao, F. Zhao, Z. Liu, S. Jiang and H. Hao, "Cradle-to-gate greenhouse gas emissions of battery electric and internal combustion engine vehicles in China," *Applied Energy,* vol. 204, pp. 1399-1411, 2017.

[31] M. V. Chester, "Life-cycle Environmental Inventory of Passenger Transportation in the United States," Institute of Transportation Studies at UC Berkeley, Berkeley , 2008.

[32] E. Helmers, J. Dietz and S. Hartard, "Electric car life cycle assessment based on real-world mileage and the electric conversion scenario," *The International Journal of Life Cycle Assessment,* vol. 22, pp. 15-30, 2017.

[33] G. Hammond and C. Jones, "Embodied Carbon - The Inventory of Carbon and Energy," BSRIA, Bracknell, 2011.

[34] Department for Transport, "Road length statistics (RDL)," 04 02 2021. [Online]. Available: https://www.gov.uk/government/statistical-data-sets/road-length-statistics-rdl. [Accessed 06 08 2021].

[35] Z. Zhang, X. Sun, N. Ding and J. Yang, "Life cycle environmental assessment of charging infrastructure for electric vehicles in China," *Journal of Cleaner Production,* vol. 227, pp. 932-941, 2019.

[36] M. Prussi, M. Yugo, L. De Prada, M. Padella and R. Edwards, "JEC Well-To-Wheels report v5," Publications Office of the European Union, Luxembourg, 2020.

[37] Department for Business, Energy and Industrial Strategy, "Government conversion factors for company reporting of greenhouse gas emissions," 2021 07 02. [Online]. Available: https://www.gov.uk/government/collections/government-conversion-factors-for-company-reporting.. [Accessed 15 09 2022].

[38] C40 Cities Climate Leadership Group, C40 Knowledge Hub, "Transport Data Explorer," 12 2020. [Online]. Available: https://www.c40knowledgehub.org/s/article/Transport-Data-Explorer?language=en_US. [Accessed 11 09 2021].

[39] R. Sinha, L. E. Olsson and B. Frostell, "Sustainable Personal Transport Modes in a Life Cycle Perspective—Public or Private?," *Sustainability,* vol. 11, no. 24, p. 7092, 2019.
